# Decidualisation and placentation defects are a major cause of age-related reproductive decline

**DOI:** 10.1038/s41467-017-00308-x

**Published:** 2017-09-05

**Authors:** Laura Woods, Vicente Perez-Garcia, Jens Kieckbusch, Xiaoqiu Wang, Francesco DeMayo, Francesco Colucci, Myriam Hemberger

**Affiliations:** 1 Epigenetics Programme, The Babraham Institute, Babraham Research Campus, Cambridge, CB22 3AT UK; 2 Centre for Trophoblast Research, University of Cambridge, Downing Street, Cambridge, CB2 3EG UK; 3 Department of Obstetrics and Gynaecology, University of Cambridge School of Clinical Medicine, NIHR Cambridge Biomedical Research Centre, Addenbrooke’s Hospital, Box 111, Hills Road, Cambridge, CB2 0SP UK; 4 Reproductive and Developmental Biology Laboratory, NIEHS, Research Triangle Park, Durham, NC 27709 USA

## Abstract

Mammalian reproductive performance declines rapidly with advanced maternal age. This effect is largely attributed to the exponential increase in chromosome segregation errors in the oocyte with age. Yet many pregnancy complications and birth defects that become more frequent in older mothers, in both humans and mice, occur in the absence of karyotypic abnormalities. Here, we report that abnormal embryonic development in aged female mice is associated with severe placentation defects, which result from major deficits in the decidualisation response of the uterine stroma. This problem is rooted in a blunted hormonal responsiveness of the ageing uterus. Importantly, a young uterine environment can restore normal placental as well as embryonic development. Our data highlight the pivotal, albeit under-appreciated, impact of maternal age on uterine adaptability to pregnancy as major contributor to the decline in reproductive success in older females.

## Abstract (web summary)

Advanced maternal age has been associated with lower reproductive success and higher risk of pregnancy complications. Here the authors show that maternal ageing-related embryonic abnormalities in mouse are caused by decidualisation and placentation defects that can be rescued by transferring the embryo from an old to a young uterus.

## Introduction

Maternal age is the single most important risk factor for reproductive success. The significance of this problem across the developed world is immense and continuously rising. Maternal age has increased steadily over the past decades; the average age of mothers surpassed 30 years for the first time in 2013 in the UK (source: ONS), with 20% of babies born to women ≥35 years of age. Very similar trends are observed in North America and elsewhere^1, 2^. The underlying cause of the increased risk to pregnancy outcome with age is commonly attributed to the exponential rise in chromosome mis-segregations in the oocyte, leading to karyotypic imbalances and aneuploidies in the offspring. Many of these karyotypic abnormalities result in spontaneous abortion in the first trimester and thus contribute to the high frequency of pregnancy loss during this time window. However, advanced maternal age also poses an increased risk of serious pregnancy complications that manifest in later pregnancy. These include miscarriage, late fetal and perinatal death, stillbirth, preterm and extreme preterm birth, low birth weight, placenta praevia and pre-eclampsia^3–10^. For example, the stillbirth rate doubles by the late 30 s and increases 3–4 fold by the mid-40s^6^. Rates of preterm birth are ~20% higher in women aged 35 years and over (source: Tommy’s; CIHI). Even beyond the gestational period, maternal age is a significant risk factor for various birth defects, in particular for congenital heart disease, and also for abnormalities including diaphragmic hernia, hypospadias and skull deformations^11–13^.

Importantly, most of these later complications occur in the absence of any chromosomal abnormality in the newborn, and as such their causative relationship to maternal age has remained largely unexplained. It is of note, however, that the majority of pregnancy complications associated with advanced maternal age, namely fetal growth restriction, low birth weight, pre-eclampsia and stillbirth, often share an underlying pathogenesis that is rooted in a failure of correct placentation^14^. Apart from the essential role of the placenta in supporting fetal growth, placental development is also tightly linked to the formation of particular organ systems, most notably the heart. This connection has been shown in a number of mouse models in which heart defects could be rescued solely by supplying the mutant embryo with a wild-type placenta^15^. Intriguingly, a recent study reported that the maternal age-associated risk of congenital heart disease resides with the mother and not the oocyte^13^. Thus, the incidence of ventricular septal defects is significantly greater in old mothers but reduced to normal levels when oocytes from old mothers are allowed to develop in a ‘young’ environment.

Based on these multi-faceted correlations, we speculated that defects in placental development may constitute a major contributor to the maternal age-associated increase in pregnancy complications, in particular those unrelated to karyotypic imbalances. To better understand the causes of maternal age-linked pregnancy complications and embryonic defects, we have used the mouse as a model to distinguish fetal (i.e. oocyte-derived) from maternal (uterine environment, systemic) effects on embryo development. Our data show that pregnancy in old female mice is associated with a dramatic increase in developmental variability, manifesting in a high frequency of developmental delays, growth retardations and serious embryonic abnormalities including cardiac, vascular and neural defects. Moreover, abnormal embryos are associated with severely mis-developed placentas. However, development of both embryo and placenta largely returns to normal when embryos from old mothers are transferred to young recipients. We pinpoint the causes of these oocyte-unrelated maternal age-associated problems to defects in the progression of decidualisation, which in turn interferes with the establishment of a functional feto-maternal exchange unit.

## Results

### Embryo and placenta defects in offspring to old females

**Fig. 1:**
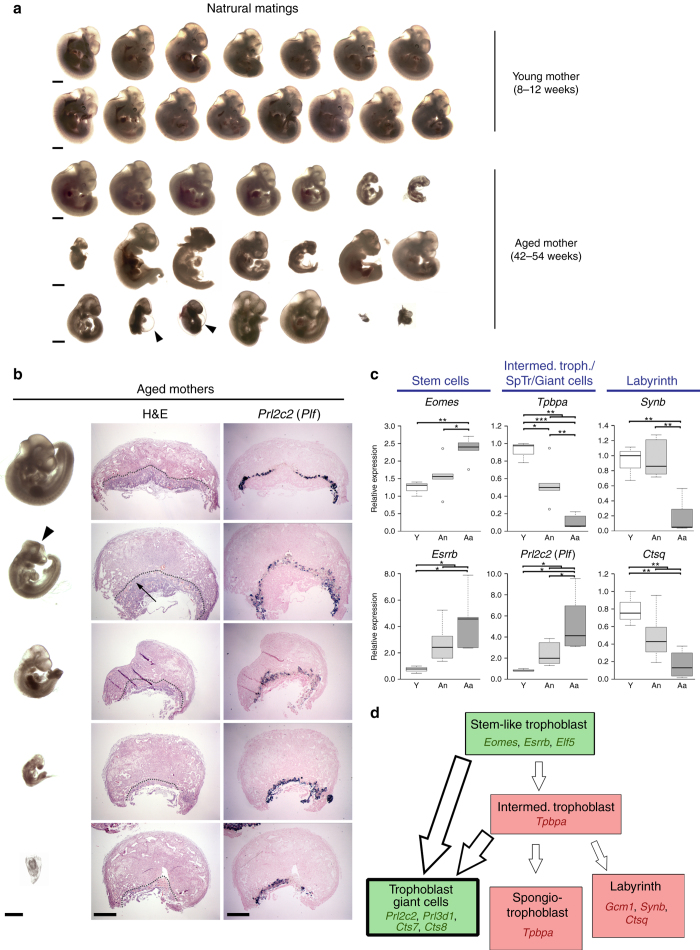
Advanced maternal age impacts on embryonic and placental development. **a** Gross morphological appearance of E11.5 embryos developed in young (8–12 weeks old) and aged (42–54 weeks old) C57BL/6 females. Each row depicts one entire litter. *Scale bars*: 1 mm. **b** Histological analysis by H&E staining and by *in situ* hybridisation for the trophoblast giant cell marker *Prl2c2* (*Plf*) of placentas associated with conceptuses developed in aged females. The *top row* depicts a normally developed embryo that is associated with a grossly normal placenta. The *four rows* below depict embryos with varying degrees of developmental retardation and morphological defects such as a failure of neural tube closure (*arrowhead*). The placentas associated with these embryos are also defective, insofar as the trophoblast portion is severely under-developed (the dotted line indicates the boundary between the fetal trophoblast compartment and the maternal decidua) or the main direction of placentation is off-centre (arrow). *Scale bars*: 1 mm. **c** RT-qPCR analysis of placentas developed in young control (“Y”, *n* = 3) and aged (“A”) females. Conceptuses developed in aged females were divided into those that appeared grossly normal (“An”; *n* = 6) or abnormal (“Aa”; *n* = 6). Markers used represent trophoblast stem cell genes *Eomes* and *Esrrb*, markers of the so-called intermediate trophoblast (as found in the ectoplacental cone) or spongiotrophoblast (SpTr) *Tpbpa* and trophoblast giant cells *Prl2c2*, and placental labyrinth expressed genes *Synb* and *Ctsq*. Data are displayed as mean ± S.E.M. *p < 0.05; **p < 0.01; ***p < 0.001 (ANOVA with Holm-Bonferroni’s post-hoc test). **d** Diagram of the main trophoblast differentiation defects frequently observed in aged females. *Green shading* indicates compartments that are relatively over-represented, whereas *red shading* depicts major differentiation routes that are missing or under-represented. Genes listed in the representative colour support these conclusions

C57BL/6 (B6) females of about 12 months of age are nearing the end of their reproductive lifespan but can still become pregnant^16^. However, it has been noted before that embryos developing in older (43–47 weeks old) C57BL/6 (B6) females are more prone to developmental abnormalities than those developing in young (6–12 week old) mothers, while the number of maturing oocytes is unaffected^17, 18^. Here, we systematically followed up on this finding by assessing a total of 40 pregnancies in B6 females ranging from 41 to 58 weeks of age. We focussed this analysis on day 11.5 (E11.5) of pregnancy, a stage in development just after mid-gestation when the consequences of failures in correct placentation become most obvious^19^. In line with previous reports, average litter size was globally similar between young and old females, albeit more variable in the aged group (Supplementary Fig. 1a). However, examination of gross morphological appearance revealed a dramatic increase in developmental variability in litters of old females compared to those of young mothers. While some embryos in litters of aged females appeared normal as far as size and developmental stage-specific hallmarks such as pigmentation of the eye were concerned, on average at least two-thirds of embryos in each litter exhibited varying degrees of developmental abnormalities (Fig. 1a and Supplementary Fig. 1b). By far the most common defect was fetal growth retardation, ranging from mild to very severe even within the same litter. Other frequent abnormalities encompassed cardiac edema, brain and neural tube closure defects, vascular defects such as a dilated dorsal aorta and/or major brain artery, as well as a significant increase in the number of resorption sites (Fig. 1a and Supplementary Fig. 1b).

Importantly, none of the litters of young females displayed such a prevalence of developmental defects. Equally, this effect was never observed in litters from young females mated with 52-week-old males (Supplementary Fig. 1b), demonstrating that the cause of the dramatic increase in developmental abnormalities resides solely within the aged mother.

Intriguingly, all these various problems in embryonic development are known to be associated with placentation defects. Therefore, we systematically analysed the placentas of aged females, alongside those of young controls, on the histological level by haematoxylin and eosin (H&E) staining, by in situ hybridisation against the trophoblast giant cell marker Proliferin (*Prl2c2*) and by expression analysis of well-established markers of the trophoblast lineage. For the latter, we grouped the placentas of litters from aged females into those associated with grossly normal embryos (“An”; *n* = 6) and those associated with overtly abnormal embryos (“Aa”; *n* = 6), and compared them to placentas from young female control litters (“Y”; *n* = 3). Overall, this analysis revealed profound defects in the placentas associated with abnormal embryos. Most frequently, the fetal trophoblast-derived portion of the placenta was drastically reduced in size (Fig. 1b). In milder cases, abnormalities in the extent and positional development of the placental labyrinth were obvious, i.e. the portion of the placenta where all nutrient and gas exchange takes place (Fig. 1b). Integrated histological and molecular data indicated a failure of appropriate differentiation of stem-like trophoblast progenitor cells, an overabundance of trophoblast giant cells at the expense of diploid (*Tpbpa* ^+^) precursor cells and a failure in development of the labyrinth layer, thus corroborating the morphological observations (Fig. 1c, d and Supplementary Fig. 1c). It is well established that such placental defects can cause embryonic phenotypes much like those observed in the litters of aged females^15, 20^. These findings were further validated by global transcriptomic analysis (RNA-sequencing (RNA-seq)) of the dissected trophoblast compartments (Supplementary Fig. 1d). We also performed an e-karyotyping analysis on the RNA-seq data using a piecewise constant fit algorithm which confirmed the absence of any gross chromosomal imbalances^21^. Interestingly, some of the differences in gene expression profiles were also observed in the “An” group, albeit to a much lesser degree, positioning the “An” samples at an intermediate stage in between the “Y” and “Aa” groups. This finding indicates that even placentas which appear grossly normal histologically, exhibit molecular changes that are likely predisposing the corresponding conceptus to developmental failures later on during pregnancy.

### Development in young females rescues most defects

Our observation of a direct correlation between abnormal embryos and placentas led us to hypothesise that the frequent developmental problems in aged females may originate in a failure to establish a functional placenta. Yet our global sequencing data, the relatively late time window of developmental failure and the recurring phenotypes indicated that these were highly unlikely to originate from karyotypic abnormalities in the oocyte. We thus considered the possibility that the cause for the perturbations in placental development may reside in the maternal aspects contributing to the placenta, notably the decidua.

**Fig. 2:**
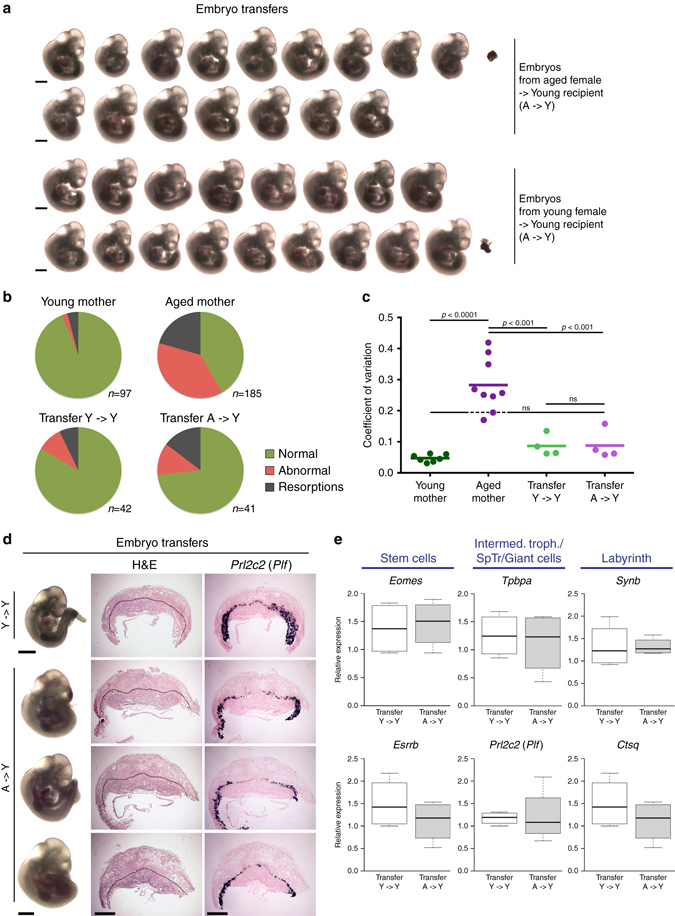
Developmental defects are mitigated by transfer of embryos from aged females into young foster mothers. **a** Litters obtained by embryo transfer from aged to young (A- > Y) recipient females, or from young to young (Y- > Y) females as controls. Note the homogeneity of developmental progression of embryos derived from aged females developing in young females, compared to the litters shown in Fig. 1a. *Scale bars*: 1 mm. **b** Pie charts of gross morphological appearance of embryos scored as normal or abnormal, and the number of resorption sites, in the different scenarios. **c** Crown-rump length measurements as indicator of developmental variability, displayed as coefficient of variation. Each data point represents the coefficient of variation of one entire litter. Please note that litters of embryos recovered from aged females that were allowed to develop in young foster mothers (Transfer A- > Y) are statistically indistinguishable from litters developed in young females (Y). Measurements were taken on E11.5 embryos. Statistical analysis was by ANOVA followed by Tukey’s multiple comparisons post-hoc test. *ns*=not significant. **d** Analysis of placental development by histology as in Fig. 1. Placentas appear normal for all embryos assessed. *Scale bars*: 1 mm. **e** RT-qPCR analysis of trophoblast markers in placentas of Y- > Y and A- > Y transfer conceptuses. Data are displayed as mean ± S.E.M., *n* = 3. No differences in expression levels are observed for any of the genes tested (Student’s *t* test), corroborating that placental development proceeds normally in A- > Y transfer conceptuses

To address this possibility, we collected preimplantation embryos at E2.5 from aged females and transferred them into pseudopregnant 8–10-week-old ‘young’ foster mothers (“A-> Y”). Transfers of embryos recovered from young females into young foster mothers (“Y- > Y”) were performed in parallel to account for technical variability. Intriguingly, providing the embryos from aged females with a young host environment in this way almost completely mitigated the developmental problems (Fig. 2a, b). The overall proportion of grossly normal embryos increased from 42% in aged females to 73% upon embryo transfer, which was only slightly below the 83% of the control Y-> Y transfers (Fig. 2b). Most strikingly, the vast majority of embryos in each litter was of similar size and developmental stage (Fig. 2a and Supplementary Fig. 2a), much in contrast to the enormous developmental variability within litters of aged females. Using crown-rump length as a proxy for developmental progression and variability, embryos from aged females allowed to develop in young foster mothers were in fact indistinguishable from young control transfer embryos (Fig. 2c).

Together with the return to a mostly normal development of the embryos proper, placental development was also rescued. Thus, histologically, placentas of A-> Y transfers were highly similar to those of Y-> Y transfers and indeed to young controls (Fig. 2d). The lack of obvious trophoblast differentiation defects was further confirmed on the level of marker gene expression (Fig. 2e and Supplementary Fig. 2b).

Taken together, our data demonstrated that the frequent developmental defects inherent to litters of older mothers were rescued by the environment of a young foster mother. Thus, the majority of these pregnancy complications was not due to defects in the “old” oocyte, but due to an unfavourable maternal environment that hampered normal developmental progression.

### Age-associated defects in decidualisation as major problem

The mammalian placenta constitutes the interface where fetal and maternal cell types meet and functionally interact to ensure normal development. The maternal component of the placenta is made up of cells of the uterine endometrium that undergo a tightly controlled process of decidualisation upon contact with the early embryo. Decidualisation involves proliferation and differentiation of the endometrial stroma into large epithelioid decidual cells, a process critical to the establishment of fetal-maternal communication and the progression of implantation. The decidualisation process has both a protective function to limit trophoblast invasion and a supportive role in placentation by producing growth factors and cytokines that help remodel the implantation site and the maternal vascular bed to promote embryo growth^22^. This process is aided by a unique leucocyte composition that forms a major fraction of the decidual compartment.

**Fig. 3:**
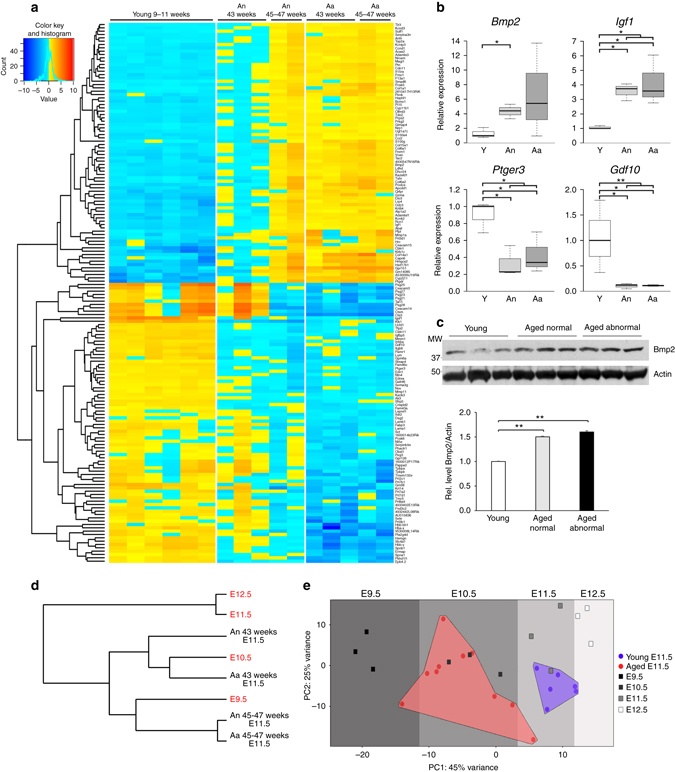
Decidual compartment is abnormal and exhibits hallmarks of developmental delay in conceptuses developed in aged females. **a** Heatmap of genes commonly de-regulated in the decidual portion of placentas developed in aged females compared to young controls. Please note that the extent of gene de-regulation increases with age of the female (43 vs. 45–47 weeks of age) and is also more pronounced in deciduas associated with abnormal embryos (Aa). Genes selected for display were significantly different between young (Y) and Aa deciduas. **b** Independent validation of differential gene expression for key decidualisation genes by RT-qPCR. Samples are as in Fig. 1c. Data are displayed as mean ± S.E.M. *p < 0.05; **p < 0.01 (*n* = 3; ANOVA with Holm-Bonferroni**’**s post-hoc test). **c** Confirmation of higher Bmp2 expression levels in deciduas developed in aged females by Western blot. Quantification of band intensities of three independent biological replicates is shown in the graph below. **p < 0.01 (ANOVA with Holm-Bonferroni**’**s post-hoc test). **d** Closest neighbour analysis of global transcriptomes generated by RNA-seq from E11.5 deciduas from aged females compared to those developed in young females of a developmental time course from E9.5-E12.5 (in *red font*). Each line represents transcriptomes of 2–9 independent biological replicates (see Supplementary Fig. 4 for all samples), with samples recovered from separate litters in every case. **e** Principal component analysis of the samples shown in **d**, showing that the E11.5 deciduas recovered from aged females exhibit an expression signature more closely resembling that of E9.5–10.5 young samples

Given that we found the majority of developmental problems in aged females to be of maternal origin, we focussed on assessing the decidual portions of placentas of the E11.5 conceptuses developed in young and aged females. To this end, we chose deciduas associated with three male and three female young control conceptuses, as well as a total of 10 deciduas of conceptuses developed in females aged 43–47 weeks, for global transcriptome analyses by RNA-seq. Interestingly, we found a cohort of 162 genes that were consistently and robustly deregulated between the young and aged groups, out of which 78 were more highly expressed and 84 down-regulated in aged females (Fig. 3a and Supplementary Data 1). These genes included key regulators of decidual differentiation, such as *Bmp2* ^23, 24^, *Gdf10* ^25^, prostaglandin D and E receptors (*Ptgdr*, *Ptger3*), as well as *Igf1* as one of the main growth factors involved in oestrogen-induced uterine growth^26, 27^. Intriguingly, using the list of differentially regulated genes for gene ontology (GO) and pathway analysis revealed a significant enrichment of genes regulated by Bmp2, as well as of growth factor-regulated pathways (“signal”, “disulphide bond”) and of extracellular matrix composition (Supplementary Fig. 3a and Supplementary Data 2). Moreover, it was apparent that the extent of deregulation of this set of genes increased with age; thus, expression profiles in deciduas from 43-week-old females were relatively more similar to controls than those from 47-week-old mothers. We independently confirmed differential expression of selected candidates by reverse transcription followed by semi-quantitative PCR (RT-qPCR) analysis (RT-qPCR) (Fig. 3b and Supplementary Fig. 3b). This included genes such as Il15 receptor alpha (*Il15ra*) as well as *Il15* (Supplementary Fig. 3b), a key cytokine of uterine natural killer (uNK) cell maturation^28, 29^. For Bmp2 as one of the most prominent decidualisation genes, we also verified its deregulated levels of abundance on the protein level by Western blot (Fig. 3c).

The observed differences in expression showed that ageing females did not simply suffer from a complete failure in decidualisation. Instead, the expression pattern of a cohort of dynamically regulated genes resembled that of an earlier developmental stage. For example, *Bmp2* and *Igf1* are highly expressed at early stages but decline later on during the decidualisation process as cells become terminally differentiated^30–32^. Conversely, *Gdf10* is up-regulated from mid-gestation to E12.5. To address this point in detail, we generated additional transcriptomes of deciduas from conceptuses developed in young control B6 females over a developmental time course between E9.5 and E12.5, and integrated them with the E11.5 datasets of the young and aged groups. Using these data for unbiased clustering and for principal component analysis confirmed that the majority of gene expression changes was determined by developmental stage (Fig. 3d, e and Supplementary Fig. 4). Thus, deciduas from aged mothers resembled an earlier developmental stage and consistently clustered more closely with the E9.5–10.5 control samples (Fig. 3d). Specifically, deciduas from 45- to 47-week-old mothers clustered closest to E9.5 control deciduas, whereas the samples from 43-week-old females clustered with the E10.5 controls. This association was evident also in those cases where the embryo still appeared grossly normal, indicating that even in these conceptuses decidualisation is altered and likely predisposes the embryo to later-onset developmental defects, resulting in the known decrease in live offspring^18, 33^. Overall, these expression patterns showed that on the global level, decidualisation is delayed in ageing females by ∼1–2 days at E11.5.

### Uterine immune cell composition is affected by maternal age

To determine the possible causes of these decidualisation defects, we first focussed on the leucocyte cell population of the pregnant uterus. An ageing environment is known to impact on NK cell maturation and function^34^. Moreover, we found a deregulated expression of the cytokine *Il15* and its receptor in aged mice, which has prominent roles in promoting NK cell survival^28^. uNK cells constitute a specialised subset of the NK cell lineage only found in the uterus where they are the most abundant immune cell type. Compared to peripheral NK cells, uNK cells exhibit reduced lytic capacity, yet they are key regulators of reproductive success. They facilitate the remodelling of maternal blood vessels and secrete angiogenic factors and cytokines, thereby influencing placentation as well as fetal growth rates^35–37^. uNK cells peak in numbers around mid-gestation when they constitute about 20% of all leucocytes in the decidua^37^. Other immune cell types at the feto-maternal interface include macrophages and dendritic cells (DCs), albeit their functions in regulating pregnancy progression are less well explored^38^.

**Fig. 4:**
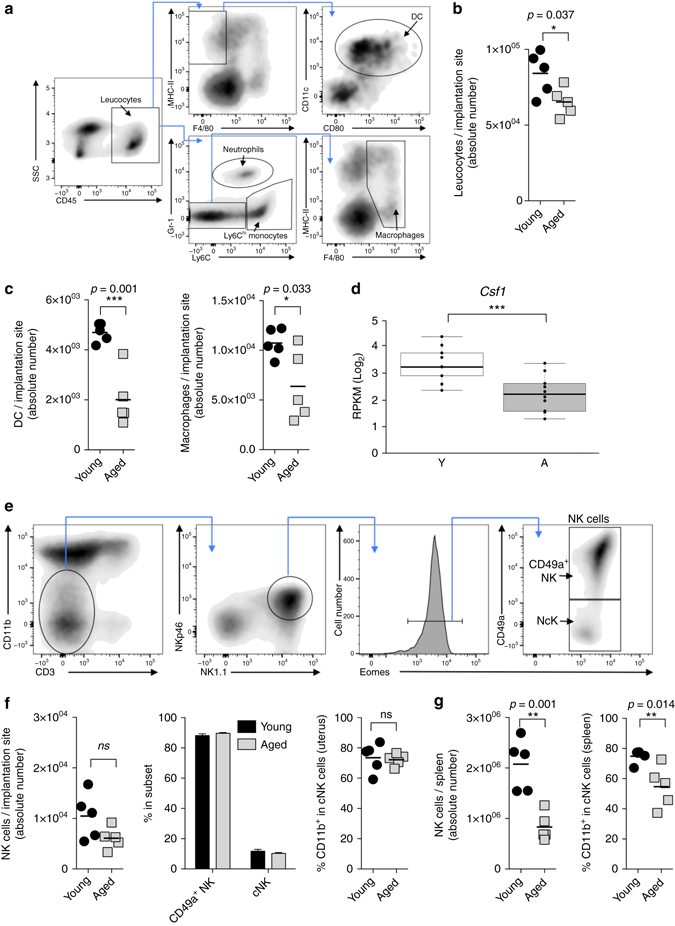
Altered leucocyte composition at the maternal-fetal interface at mid-gestation. **a** Flow cytometry analysis of decidual leucocytes: gating strategy showing delineation of uterine cells into macrophages and dendritic cells (DCs, pre-gated on live singlets). **b** Absolute number of leucocytes recovered per implantation site. **c** Enumeration of macrophages and DCs at the maternal-fetal interface. **d** Expression levels of *Csf1*, a key cytokine of DC cell maturation, as determined by RNA-seq, displayed as normalised read counts of 9 (young = Y) and 10 (aged = A) independent replicates. Data are displayed as mean ± S.E.M. ***p < 0.001 (two-tailed *t* test). **e** Gating strategy used to identify uNK cells and delineation into tissue-resident CD49a^+^ and conventional CD49a^−^ cells (pre-gated on live singlets). **f** Enumeration of NK cells per implantation site and relative abundance of tissue-resident and conventional uNK cells, as well as frequency of more mature CD11b^+^ uNK cells. **g** Enumeration and maturation phenotype of peripheral splenic NK cells in pregnant females. Each data point in **b**, **c**, **f** and **g** represents an entire litter of one pregnant mouse, values in **f** are means ± S.E.M

In order to determine whether maternal age has an impact on composition and/or maturation of immune cell populations at the feto-maternal interface, we devised a detailed flow cytometry strategy to delineate macrophage, DC and uNK cell populations (Fig. 4a). Overall, we observed a slight reduction in the total number of leucocytes in the aged deciduas (Fig. 4b). This decrease was mainly driven by a reduction in macrophage and DC numbers (Fig. 4c) and correlated with lower expression levels of *Csf1* (Fig. 4d), a key regulatory cytokine of macrophages and DCs in the uterus^38^. By contrast, we did not detect differences in the abundance of uNK cells, neither for the tissue resident CD49a^+^ subset nor the conventional NK cell subset (Fig. 4e, f and Supplementary Fig. 5). Furthermore, both NK cell subsets displayed a normal maturation phenotype in aged mice as assessed by staining for CD11b (Fig. 4f). This is in contrast to peripheral NK cells in the spleens of aged females where a marked reduction in NK cell numbers is observed, particularly in the more mature CD11b^+^ subset (Fig. 4g), as previously reported^39^. Taken together, these results show that ageing leads to an overall reduction in leucocyte numbers at the maternal-fetal interface. However, uNK cell abundance or maturation is not affected, suggesting that the pregnant uterus is protected from the systemic NK cell decline during ageing.

### Aged uterine stromal cells are refractory to decidualisation

**Fig. 5:**
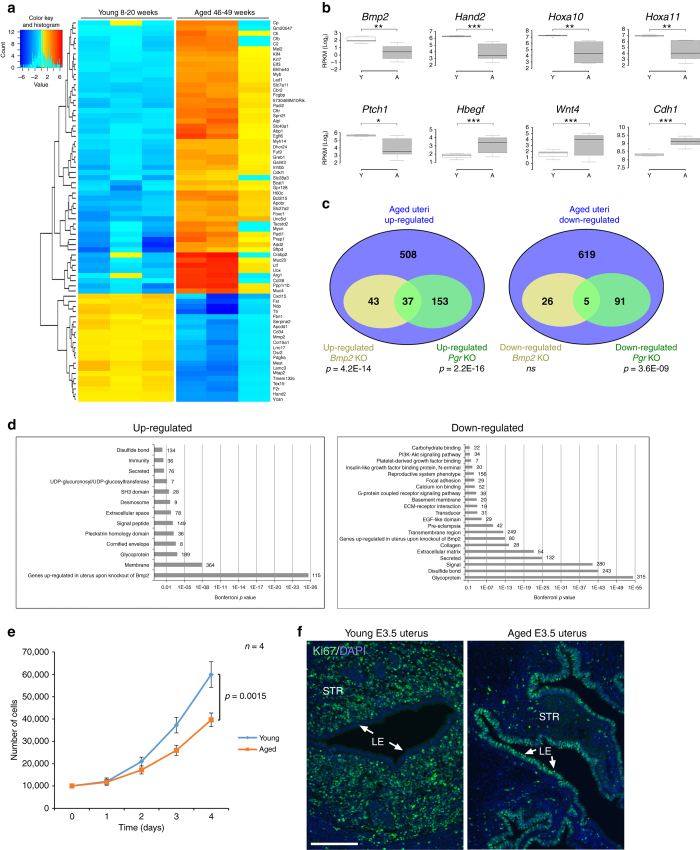
Decidualisation defects are evident in the pre-receptive uterus. **a** Heatmap of differentially expressed genes in E3.5 uteri of young and aged females that were mated with vasectomised males. Two independent samples per uterus dissected from corresponding regions were assessed per animal, resulting in six sequencing samples from a total of three animals in each age group. **b** Examples of key regulatory genes in decidualisation that exhibit significantly divergent expression between uteri of young and aged females. Values are normalised for total read counts and displayed as mean ± S.E.M. (*n* = 3). *p < 0.05; **p < 0.01; ***p < 0.001 (two-tailed *t* test). **c** Venn diagrams of genes commonly up-regulated or down-regulated in uteri of aged E3.5 females and in knockouts for *Bmp2* and *Pgr*. **d** Gene ontology and enrichment analyses of genes differentially up-regulated or down-regulated between E3.5 uteri of young and aged females. **e** Proliferation assay of isolated uterine stromal cells on 4 consecutive days after plating. Cells from aged females exhibit significant proliferation defects (mean ± S.E.M., *n* = 4). Two-way ANOVA with Holm-Sidak**’**s multiple comparisons test. **f** Ki67 staining of E3.5 uteri of young and aged females. *Arrows* point to luminal epithelium. STR = stromal cell compartment, LE = luminal epithelium. *Scale bar*: 200 µm

To corroborate the view of a more prominent defect in stromal vs. uNK cells, we interrogated our transcriptomics data against a recently identified gene expression signature of uNK and decidual stromal cells^40^. Indeed we found that the stromal cell genes separated the young from the aged samples more clearly than the uNK cell genes (Supplementary Fig. 6a). Our next approaches were aimed at refining this analysis so as to trace any differences in uterine responses back to the very early steps of pregnancy and independent of embryo-induced influences. For this purpose, we mated young and aged females with vasectomised males and dissected equivalent pieces of the uterine horns at E3.5 for transcriptomic analysis. Although there was some variability between the aged females, it was evident that critical initiators of decidualisation were already de-regulated at this early stage (Fig. 5a and Supplementary Data 3). Notably, inspection of candidate genes revealed that *Bmp2*, *Hand2*, *Hoxa10*, *Hoxa11*, *Ptch1*, *Areg* and *Foxa2* were down-regulated in E3.5 uteri of aged females even in the absence of the stimulus by an implanting blastocyst, while *Wnt4*, *Hbegf* and E-Cadherin (*Cdh1*) were up-regulated (Fig. 5b)^23, 41–46^. Comparing our data to the molecular signatures of knockouts for *Bmp2*, progesterone receptor (*Pgr*), *Egfr* and *Wnt4*, all of which exhibit profound decidualisation defects, showed significant overlaps between de-regulated gene sets (Fig. 5c, Supplementary Fig. 6b and Supplementary Data 4). Global functional annotation analyses indicated that dysregulated genes were enriched for factors in glycoprotein and secreted protein repertoire, extracellular matrix composition, membrane components and Bmp2-regulated factors (Fig. 5d and Supplementary Data 2). It is noteworthy, however, that the abundance of growth factors with critical roles in the decidualisation response, chiefly *Lif* and *Egf*, remained unchanged as did their receptors *Lifr* and *Egfr* ^47, 48^.

As Bmp2 has been previously shown to affect proliferation during decidualisation^23^, we tested the proliferative capacity of isolated E3.5 uterine stromal cells in vitro and found that those from aged females exhibited dramatically decreased levels of cell proliferation (Fig. 5e). These data were further confirmed on the histological level by Ki67 staining of E3.5 uteri. Here, too, the uterine stroma of aged mice exhibited a low proliferative activity compared to young controls (Fig. 5f). However, high levels of Ki67-positive staining was retained in the luminal epithelium, indicating that the epithelial-to-stromal cell switch in proliferative activity in response to progesterone had not taken place yet in the majority of aged samples^49^.

### Blunted hormone responsiveness hampers decidualisation

**Fig. 6:**
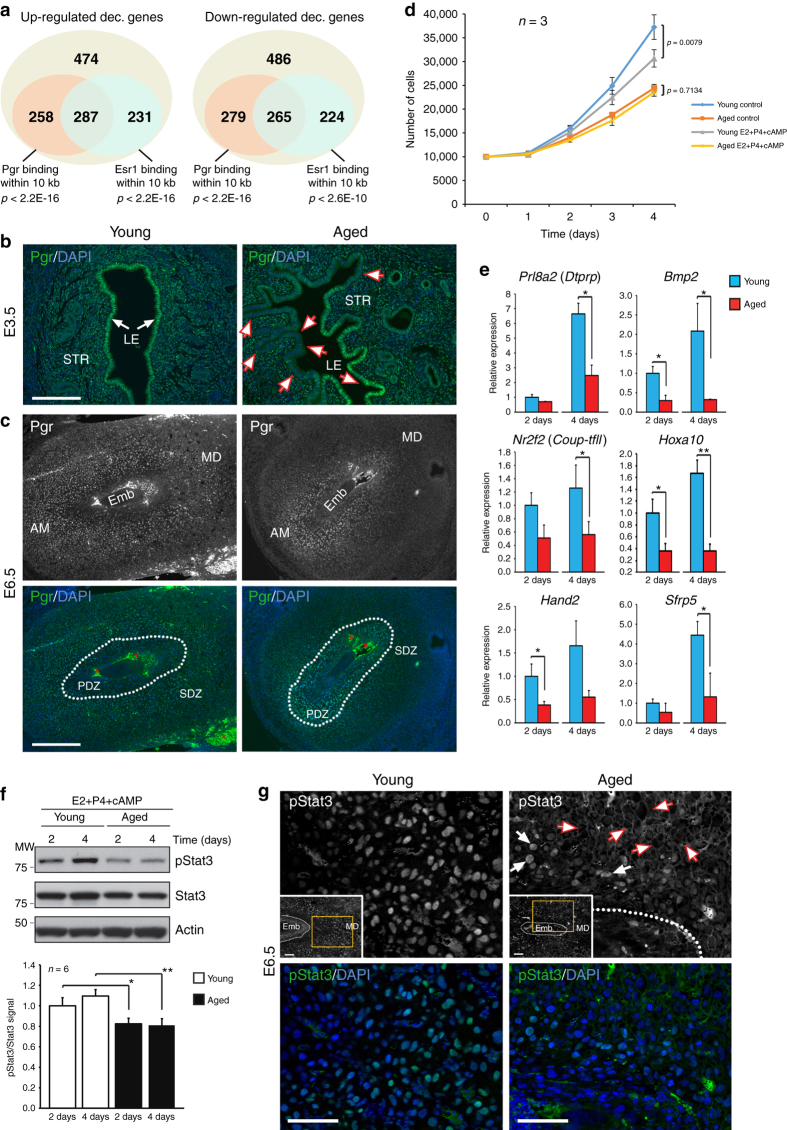
Blunted hormone responsiveness in uterine stromal cells of aged females. **a** Venn diagram of de-regulated genes in deciduas of aged females and their proximity to oestrogen receptor-α (Esr1) and progesterone receptor (Pgr) binding elements. **b** E3.5 uteri stained for Pgr. White arrows point to the homogenous staining of Pgr in luminal epithelium (LE) of young uteri. *Red-lined arrows* highlight the mosaic staining of Pgr in LE of aged uteri with large patches exhibiting drastically reduced Pgr levels. *Scale bar*: 200 µm. **c** E6.5 implantation sites developed in young and aged females stained for Pgr. *Dotted lines* demarcate the boundary between the primary (PDZ) and secondary (SDZ) decidualisation zones. In young females, decidualisation has progressed such that Pgr staining is strongest in the SDZ, whereas in aged females staining still highest in the PDZ. *Red asterisks* demarcate autofluorescence from blood cells. *Scale bar*: 500 µm. **d** Proliferation curve of decidual stromal cells isolated from young and aged females either unstimulated or after exposure to the decidualisation-inducing cocktail estrogen (E2), progesterone (P4) and cAMP (mean ± S.E.M., *n* = 3). Two-way ANOVA with Holm-Sidak’s multiple comparisons test. **e** RT-qPCR analyses of decidualisation markers in uterine (decidual) stromal cells stimulated for 2 and 4 days (mean ± S.E.M., *n* = 3). *p < 0.05; **p < 0.01 (pairwise one-tailed *t*-test). **f** Western blot for phosphorylated Stat3 (pStat3) in stimulated uterine stromal cells of young and aged females. Graph shows the pStat3/Stat3 ratio normalised to 2 day young cells (mean ± S.E.M., *n* = 6). *p < 0.05; **p < 0.01 (ANOVA with Holm-Bonferroni**’**s post-hoc test). **g** pStat3 staining of E6.5 implantation sites. Insets show overview photographs of the implantation sites (Emb = embryo, MD = mesometrial decidua), the *yellow rectangles* demarcate the magnified areas depicted. In young females, pStat3 staining is confined to the nuclei of decidualised stromal cells, indicative of its functionally activated state including as co-activator of Pgr-responsive genes. In aged females, only very few decidual cells exhibit nuclear pStat3 staining (*white arrows*), whereas in the majority of decidual cells (*red-outlined arrows*) staining is distinctly excluded from the nucleus. *Scale bars*: 100 µm

Gene expression programmes associated with the earliest steps of decidualisation are governed by the pregnancy hormones oestrogen (E2) and progesterone (P4)^50^. The de-regulation of critical drivers of decidualisation, such *Bmp2* and *Hoxa10*, as well as the observed delay in the switch from epithelial to stromal cell proliferation were strong indicators that uteri of aged mice exhibit problems in their hormonal responsiveness. Assessing the cohort of genes de-regulated in deciduas of conceptuses developed in aged females indeed revealed that over 50% of them are associated with an oestrogen receptor-α (Esr1) and/or Pgr binding site^51–53^, a far greater number than expected by chance (Fig. 6a). Although global expression levels of Pgr, specifically the Pgr-A isoform that is dominant for uterine function^54^, were not consistently changed in stromal cells of aged females (Supplementary Fig. 6c, d), we noted striking differences in tissue-specific distribution. Intriguingly, expression of both Pgr and Esr1 receptors, as well as the phosphorylated active form pEsr1, was more variable in aged uteri. This was particularly evident in the luminal epithelium where a highly mosaic staining pattern was observed (Fig. 6b and Supplementary Fig. 6e). At E6.5, strongest Pgr staining had progressed to the secondary decidual zone in young females. In aged samples, by contrast, highest levels were still confined to the primary decidual zone in close proximity to the conceptus, further corroborating a slower decidualisation response in older females (Fig. 6c).

Since implantation *per* se was not impaired in aged females but decidualisation of the stromal compartment appeared delayed, we focussed on studying the progression of decidualisation in response to the hormonal stimulus more closely in isolated uterine stromal cells *in vitro*. When exposed to the well-established cocktail of E2, P4 and cAMP to trigger decidualisation, stromal cells from young females slowed down in proliferation as they started to differentiate (Fig. 6d). By contrast, stromal cells from aged females—which already exhibited much reduced proliferation rates under unstimulated conditions—did not change upon exposure to the hormone cocktail, thus underpinning a significant defect in the response to the hormonal stimulus (Fig. 6d). To further elucidate the hypo-responsiveness of aged cells to the decidualisation trigger, we collected cells after 2 and 4 days of exposure to the hormone cocktail, and assessed a panel of decidualisation markers. Uterine stromal cells from aged mice exhibited significantly lower gene expression levels of *Prl8a2* (*Dtprp*), *Bmp2*, *Hand2*, *Hoxa10*, *Nr2f2* (*Coup-tfII*), *Igfbp5* and *Sfrp5* compared to young stromal cells (Fig. 6e and Supplementary Fig. 7a). These data indicate that the aged cells exhibit an intrinsic defect in the temporal progression of decidual differentiation.

To pinpoint possible causes of the reduced decidualisation response, we analysed the signal transducer and activator of transcription-3 (Stat3). Activated Stat3 (pStat3) synergises with Pgr by physically interacting with the Pgr-A isoform; as such, Stat3 is required for the onset of decidualisation and appropriate activation of Pgr target genes^55–57^. We therefore assessed the levels of pStat3 in stromal cells from young and aged mice, and consistently found reduced levels of pStat3 in the hormone-stimulated cells of aged females (Fig. 6f and Supplementary Fig. 7b). More significantly even, the subcellular distribution of pStat3 was dramatically altered in stromal cells of aged E3.5 uteri and E6.5 implantation sites. While activated Stat3 signalling was evident by prominent nuclear pStat3 staining in the young samples, pStat3 was excluded from the vast majority of stromal cell nuclei in aged females (Fig. 6g and Supplementary Fig. 7c). Since Stat3 phosphorylation is induced by Lif signalling through Lifr via the Jak/Stat pathway, we tested whether addition of exogenous Lif to stromal cell cultures could ameliorate the decidualisation defect; however, the presence of Lif did not accelerate the progression of decidualisation of aged stromal cells, thus further supporting the cell-intrinsic nature of the defect (Supplementary Fig. 7d). Thus, our data demonstrate that maternal age impacts on the hormonal responsiveness of uterine stromal cells by interfering with the availability of activated Stat3 as coactivator of the Pgr response, which interferes with appropriate target gene activation.

## Discussion

The negative impact of maternal age on reproductive outcome is most commonly associated with the exponential increase in non-disjunctions and other meiotic defects in the oocyte. What has remained much less appreciated is the increase in reproductive problems that have no karyotypic basis. In humans these manifest as various pregnancy complications such as pre-eclampsia, stillbirth or fetal growth restriction, as well as a range of developmental defects to the newborn. Similarly in mice, it has been noted that the number of live offspring declines in older females in the absence of any appreciable depletion in oocyte stores and despite unchanged numbers of early implantation sites^18, 33^. Thus, embryo loss must occur post-implantation, as evidenced by a higher frequency of resorptions sites and developmental delays at mid-gestation. It has also been noted that placental development is perturbed by female ageing^17^. Here, we systematically follow up on this observation and find that the extent of developmental variability increases dramatically in females aged 40 weeks and older. More significantly, we find that abnormal embryos are associated with abnormal placentas in every case assessed, suggesting that the malfunctional placenta may indeed be the primary cause of embryonic demise. Taking this thought further, it is even tempting to speculate that the increased risk of congenital heart defects that resides with the older mother may in fact be caused by failures in correct placentation^13^.

Strikingly, the entire range of placental and embryonic abnormalities can be rescued when fertilised embryos recovered from aged females are allowed to develop in young foster mothers. Our data further demonstrate that the critical age-associated problems reside in the uterine response to pregnancy that in turn interferes with normal development of the placenta. In line with this, early studies showed that the reverse transfer of embryos from young donors into old recipients hampers developmental progression, even though these experiments are technically challenging^58^. Indeed, changes to the uterus as a likely cause of the fewer offspring to aged females have been hinted at before^18^, perhaps associated with an increase in collagen fibres^59^. Concordant with these early observations, our transcriptome analyses revealed extracellular matrix components as a significantly de-regulated pathway in aged deciduas. Yet there is no evidence from mouse models that these factors alone cause the rather dramatic abrogation of developmental progression observed in aged females. What then are the age-related changes to the decidualising uterus that interfere with placental development?

To address this question, we systematically dissected the impact of age on the two major cellular components of the decidua, i.e. uterine-specific leucocytes and decidual stromal cells. As far as immune cell types are concerned, there is indeed evidence that an ageing micro-environment compromises maturation and function of peripheral NK cells^34^. Consistent with these findings, we observe reduced NK cell numbers in the spleens of aged females. Yet in the mid-gestation decidua, the uNK cell population remains largely unchanged both in terms of total cell number and maturation state between implantation sites developed in young and aged females. By contrast, we observed a drastic reduction in the number of DCs, which is associated with reduced expression levels of *Csf1* as a critical cytokine directing their recruitment and maturation^38^. DCs direct the immune response at the implantation site in the presence of the semi-allogeneic fetus^60^. Moreover, the depletion of DCs just before implantation has been shown to disrupt embryo implantation and decidualisation. Indeed it is associated with impaired proliferation and differentiation of endometrial stromal cells^61, 62^. Thus, the reduction of DCs and their trophic function in the decidualised uterus of aged mice may well be an important contributory factor to the perturbations of placental development.

Collectively, however, our data point to a more profound defect in the stromal cell compartment. The majority of genes that are de-regulated in aged deciduas form part of the transcriptional programmes directed by the Pgr and Esr1 receptors^51, 53^. Consistent with this, we noted a striking heterogeneity in expression of both receptors already at E3.5 in the uterine epithelium of aged females. It is tempting to speculate that this pattern may indeed explain the spread in phenotypes that is characteristic of pregnancy in aged female mice. Conceptuses that appear normal at mid-gestation may have implanted at sites of high Pgr and Esr1 expression, whereas severely growth-retarded implantations may have occurred at Pgr/Esr1-depleted sites. The developmental kinetics of global expression profiles further showed that decidualisation was delayed by 1–2 days at E11.5. We demonstrate that this delay can be traced back to the very early stages of hormonal priming of the uterus and that it is independent of embryo-induced effects. Stromal cells of aged females exhibit significantly reduced proliferation rates and are blunted in their molecular response to the decidualisation stimulus. These findings corroborate and extend previous reports that deciduomata from old mice are smaller than those from young^63^. They also show that the impairment is independent of hormone levels *per* se, as cells *in vitro* were exposed to identical culture conditions.

Integrating our data with other models of decidualisation failure, we find significant overlaps with master regulators of decidualisation, notably factors in the Pgr pathway including Bmp2, Nr2f2 and Ptch1. In line with this, Pgr-mediated effects such as the down-regulation of *Ltf*, *Muc1* and *Cdh1* are less efficient or delayed with age, as evident from our transcriptional profiles (Supplementary Data 3), even though this does not impact on implantation rates^18, 33, 47^. Yet other factors, such as the steroid receptor co-activators *Ncoa1* and *Ncoa2*, that help to relay the Pgr signal in the nucleus, as well as Lif availability remain seemingly unchanged, at least on the transcriptional level^64^. Importantly, however, we demonstrate that the overall abundance and specifically the nuclear availability of activated Stat3 is profoundly reduced in stromal cells of aged females. Given that pStat3 binds to and synergises with Pgr to achieve adequate levels of target gene activation, it is most likely that this reduction in transcriptional activator availability underlies the inability to elicit an appropriate decidualisation response. Indeed, selective deletion of Stat3 from stromal cells has been reported to result in a significant reduction in embryonic survival, increased resorption rates and placental dys-morphology^65^. Interestingly, Stat signalling was also predicted as a key transcriptional regulatory pathway affected by age in the non-pregnant myometrium, thus lending further support to our findings^66^.

Overall, our study highlights the importance of the ageing uterine environment as a prevalent cause of reproductive decline in older females. Although this concept has been noted to some extent before^58, 67^, the underlying molecular changes had remained elusive. In general, the considerable impact of maternal age on reproductive success unrelated to oocyte fitness has not been widely appreciated. Even though it remains to be established to what extent these findings in the mouse translate to the situation in humans, our data prompt an in-depth investigation into the impact of maternal age on the capacity of the reproductive tract to support normal pregnancy unrelated to oocyte health.

## Methods

### Mice

C57BL/6Babr mice were used throughout this study. Virgin females were caged in groups up to 5 until they had reached the desired age as indicated. All animal experiments were conducted in full compliance with UK Home Office regulations and with approval of the local animal welfare committee (AWERB) at The Babraham Institute, and with the relevant project and personal licences in place. “Young” females were generally 8–12 weeks old. Timed matings were set up with standard C57BL/6Babr stud males of 8–16 weeks of age, counting the morning of the vaginal plug as E0.5. “Aged” males were 1 year old. Pregnant females were dissected at the gestational age indicated, and processed for histology, RNA isolation or stromal cell isolation as described.

### Histology

Placentas of E11.5 conceptuses, E3.5 uteri and E6.5 whole implantation sites were fixed overnight in 4% paraformaldehyde and processed for routine paraffin histology. Sections of 7 µm were cut on a Leica paraffin microtome. For H&E) staining, sections were deparaffinised and incubated in Mayer’s haematoxylin (Sigma #51275) for 10 min, followed by a brief wash in tap water. Differentiation of staining was carried out in 70% acid alcohol (70% ethanol, 1% hydrochloric acid) for 10 s, followed by incubation in cold tap water for 10 min. Eosin (alcoholic eosin, Sigma #HT110116) staining was performed for 30 s, followed by dehydration of sections and mounting with Eukitt quick-hardening mounting medium (Sigma #03989).

For immunostainings, antigen retrieval was performed by boiling in 1 mM EDTA, pH 7.2, 0.05% Tween-20 or in 10 mM Na citrate buffer, pH 6.0, followed by blocking in PBS, 0.5% BSA and 0.1% Tween-20. Antibodies used were Ki67 (Abcam ab15580, 1:200 dilution), Pgr (Dako A0098, 1:1000), pStat3 (Cell Signaling 9138S, 1:200), Esr1 (Santa Cruz sc-543, 1:200) and pEsr1 (Santa Cruz sc-101675, 1:200). Secondary antibodies used were either the appropriate AlexaFluor or horseradish peroxidase-conjugated antibodies used at 1:500 and 1:100, respectively. uNK cell staining was performed with horseradish peroxidase-conjugated *Dolichus biflorus* agglutinin (Sigma #L1287) at 1:100 dilution following boiling in 0.01 M Na citrate buffer, pH 6.0. Counter-staining was performed with 4′,6-diamidino-2-phenylindole for immunofluorescence and hematoxylin for immunohistochemistry.

For RNA in situ hybridisations, linearised plasmids containing cDNA inserts from the *Plf* cDNA were used to generate digoxigenin (DIG)-labelled riboprobes using the DIG RNA-labelling protocol according to the manufacturer’s instructions (Roche #11277073910). In situ hybridisations were carried out at 52 °C overnight using standard procedures. Signals were detected with anti-DIG-alkaline phosphatase-conjugated antibody (Roche), and staining was performed overnight using NBT and BCIP reagents (Promega #S3771). Sections were counterstained with nuclear fast red (Sigma #N3020).

### RT-qPCR expression analysis

For RT-qPCR analysis, total RNA was extracted with TRI reagent (Sigma T9424). Total RNA of 0.5–1 µg was reverse transcribed with random hexamer primers (Thermo Scientific SO142) using RevertAid H-Minus reverse transcriptase (Thermo Scientific EP0451) according to the manufacturer’s instructions. qPCR was performed using SYBR Green Jump Start Taq Ready mix (Sigma S4438) on a Bio-Rad CFX96 or CFX384 thermocycler using gene-specific intron-flanking primers (Supplementary Table 1). Gene expression was analysed on triplicate samples and the Ct values were normalised to *Sdha* housekeeping gene. Where appropriate, Student’s *t* tests or analysis of variance (ANOVA) were performed to calculate statistical significance of expression differences (*p* < 0.05). mRNA expression is expressed as the mean relative to the control sample; error bars indicate standard error of the mean (S.E.M.). Box plots were generated using http://shiny.chemgrid.org/boxplotr/.

### RNA-seq

For RNA-seq from deciduas, total RNA was prepared using Allprep DNA/RNA Mini kit (Qiagen 80204) followed by Dnase treatment using the TURBO DNA-free kit (Life Technologies AM1907). mRNA was isolated from total DNA-free RNA (350 ng) using the Dynabeads mRNA purification kit (Life Technologies 61006). Indexed, strand-specific libraries were prepared using the ScriptSeq v2 RNA-Seq Library Preparation Kit (Epicentre SSV21106) according to manufacturers’ instructions. Libraries were quantified/assessed using both the KAPA Library Quantification Kit (KAPA Biosystems KK4824) and Bioanalyzer 2100 system (Agilent). Indexed libraries were pooled and sequenced with a 100 bp single-end protocol on an Illumina HiSeq2500 sequencer. Raw fastq data were mapped to the *Mus musculus* GRCm38 genome assembly using TopHat v2.0.12.

### Bioinformatic analysis

Data were quantitated at a protein-coding mRNA level using the RNA-seq quantitation pipeline in SeqMonk software (http://www.bioinformatics.babraham.ac.uk), and normalised according to total read count (reads per kilobase of transcript per million mapped reads, RPKM), and 75% distribution. Differential expression was calculated using DESeq2 and intensity difference in SeqMonk, with a *p* value threshold of 0.05 and adjusted for multiple testing correction using the Benjamini-Hochberg method. Stringently differentially expressed genes were defined as meeting both DESeq2 and intensity difference criteria, whereas less stringent genes met one or both criteria.

Heatmaps were generated using Heatmap.2 in R. Principal component analysis was performed using DESeq2 rlog-normalised RNA-seq data on read counts using the top 500 most variable genes, before plotting the principal component analysis using prcomp in R. GO and gene set enrichment analyses using the MSigDB database (http://software.broadinstitute.org/gsea/msigdb) were performed on genes found to be significantly differentially up-regulated or down-regulated, separately, against a background list of genes consisting of those with more than 20 reads aligned. GO terms with a Bonferroni *p* value of <0.05 were found using DAVID^68^, Gorilla^69^ and GREAT GO resources. Venn diagrams were plotted using BioVenn http://www.cmbi.ru.nl/cdd/biovenn/.

For the analysis of gene proximity to Pgr and Esr1 binding elements, we first determined the cohort of differentially expressed genes between young and aged deciduas using DESeq2 and intensity difference in SeqMonk. Proximity of these genes to Pgr and Esr1 binding sites^51–53^ was determined using published data, available in NCBI GEO dataset accession numbers GSE34927 and GSE36455 in bed format, using a 10 kb cut-off. Differentially expressed genes were split into up-regulated and down-regulated genes, which were analysed separately. Statistical significance of the overlap was calculated by Fisher’s exact test in R.

Microarrays of uterine RNA samples from conditional ablation of *Bmp2*, *Wnt4*, *Egfr2* and their respective control groups were performed by Genomic and RNA profiling Core of Baylor College of Medicine as described previously^45^. Two-tailed *t* test and fold changes were used to define differentially expressed probes. To obtain a non-redundant list of significantly altered genes, multiple probe sets for a given gene (when present) were averaged. Genes with an absolute fold change of ≥1.4 and a *p* value of ≤0.01 were considered for further analysis. RNA-seq data from E3.5 uteri were split into up-regulated and down-regulated genes, and compared with the microarray datasets. The significance of the pairwise overlap between the datasets was determined by Fisher’s exact test, against a background of all genes detected in both RNA-seq and microarray datasets.

### Stromal cell isolation and culture

Fresh stromal cell isolation as previously described^70^ with a few modifications. In brief, uteri from E3.5 mice were slit longitudinally and disaggregated with 2.5% pancreatin (Sigma P3293) and 0.5% trypsin (type III) (Sigma T4799) in Hank’s basic salt solution (HBSS)-calcium/magnesium free for 1.5 h on ice. After vortexing, the medium was removed and the remaining tissue washed twice in HBSS (discarding luminal epithelial cells) and incubated 30 min at 37 °C in HBSS containing 0.007% collagenase (ThermoFisher Scientific 17104-019), 0.02% DNase I (Roche 10104159001), 0.008% protease (Sigma P8811) with vortexing every 5 min. Dissociated tissues were then triturated, filtered through a 70 μm cell strainer (BD Biosciences) and spun at 1000 × *g* for 5 min at 4 °C. Cell pellets were resuspended in phenol-red free Dulbecco’s modified Eagle’s medium: Nutrient Mixture F-12 (DMEM/F-12) (ThermoFisher Scientific 21041-025) plus 10% foetal bovine serum, 1 mM sodium pyruvate (ThermoFisher Scientific 11360-039), 1X antimycotic/antibiotic (ThermoFisher Scientific #15240-062) and 50 µM 2-mercaptoethanol (ThermoFisher Scientific #31350-010).

Decidualisation of cultured uterine stromal cells was induced by incubating the cells with complete DMEM/F-12 medium plus 10 nM β-estradiol (Sigma #E2758), 1 µM medroxyprogesterone 17-acetate (Sigma #M1629) and 10 µM 8-bromoadenosine 3′,5′-cyclic monophosphate (Sigma #B5386) (E2 + P4 + cAMP). The culture medium was changed every 2 or 3 days with continuous supplementation with these treatments.

### Proliferation assay

For analysis of cell proliferation rates, 10,000 uterine stromal cells from young and aged mice were plated in either complete DMEM/F12 medium or complete medium containing the decidualisation-inducing cocktail (E2 + P4 + cAMP) and collected every 24 h over 4 days. After trypsinisation the number of viable cells was counted using the Muse Count & Viability Assay Kit (Merck Millipore MCH100102) and run on the Muse cell analyser (Merck Millipore), according to manufacturer’s instructions. Statistical analysis was performed using ANOVA followed by Holm-Sidak’s post-hoc test.

### Western blot

Total cell extracts from deciduas were prepared in radioimmunoprecipitation assay buffer (20 mM Tris-HCl, pH 8.0, 137 mM NaCl, 1 mM MgCl_2_, 1 mM CaCl_2_, 10% glycerol, 1% NP-40, 0.5% sodium deoxycholate, 0.1% sodium dodecyl sulphate), or in the case of uterine stromal cells, cells were lysed in a detergent buffer (10 mM Tris-HCl, pH 7.4, 150 mM NaCl, 10 mM KCl, 0.5% Nonidet P-40) containing a protease inhibitor cocktail (Sigma P2714), and incubated at 4°C for 1 h, followed by centrifugation (9300 × *g*, 10 min). Western blotting was performed following a standard protocol. Blots were probed with the following antibodies: anti-pStat3 (Tyr705) (Cell Signaling #9138S), anti-Stat3 (Cell Signaling #12640S), anti-Pgr (Dako #A0098), anti-beta-actin (Abcam ab6276), anti-Bmp2 (Peprotech #500-P195). Horseradish peroxidase-conjugated secondary antibodies were from Bio-Rad. Detection was carried out with enhanced chemiluminescence reaction (GE Healthcare RPN2209) using standard X-ray films. Band intensities were quantified using ImageJ software. All primary scans of Western blots are provided in Supplementary Fig. 8.

### Leucocyte analysis

Preparation of single cell suspensions and FACS analysis were performed as per established protocols. Briefly, mesometrial poles of E10.5 implantation sites were dissected, pooled and minced and tissue homogenates digested with Liberase TM (Sigma 05401119001). Cells were typed with fluorochrome-conjugated antibodies for CD45 (30-F11), NK1.1 (PK138), CD11b (M1/70), CD49a (HMα1), CD49b (DX5), I-A/I-E (M5/114.15.2), Gr-1 (RB6-8C5), F4/80 (BM8), CD11c (N418), CD3ε (145-2C11), NKp46 (29A1.4) and Eomes (Dan11mag) purchased from BD PharMingen, BioLegend or eBioscience. For intracellular and intranuclear antigens, Fix & Perm and Foxp3 staining buffer set (both eBioscience) were used, respectively. Cells were acquired on an LSRFortessa.

### Data availability

All primary data are available from the authors upon request. Genome-wide sequencing data are deposited in the GEO database under accession number GSE98901; they are also available directly from the authors.

## Electronic supplementary material

Supplementary Data 1

Supplementary Data 2

Supplementary Data 3

Supplementary Data 4

Supplementary Information
