# Supplementary material for: Decidualisation and placentation defects are a major cause of age-related reproductive decline
Source: Nat Commun. 2017 Sep 5;8:352. doi: 10.1038/s41467-017-00308-x (PMC5585348; doi:10.1038/s41467-017-00308-x)
Supplement: Supplementary file 5 — Supplementary Information [file 41467_2017_308_MOESM5_ESM.pdf]

Title: Supplementary Information

Description: Supplementary Figures, Supplementary Table, and Supplementary References

Title: Supplementary Data 1

Description: List of differentially expressed genes in E11.5 deciduas of young and aged females.

Title: Supplementary Data 2

Description: Detailed gene ontology term analysis results.

Title: Supplementary Data 3

Description: List of differentially expressed genes in E3.5 uteri of young and aged females.

Title: Supplementary Data 4

Description: Genes commonly de-regulated in Bmp2 and Pgr knockouts and uteri of aged females.

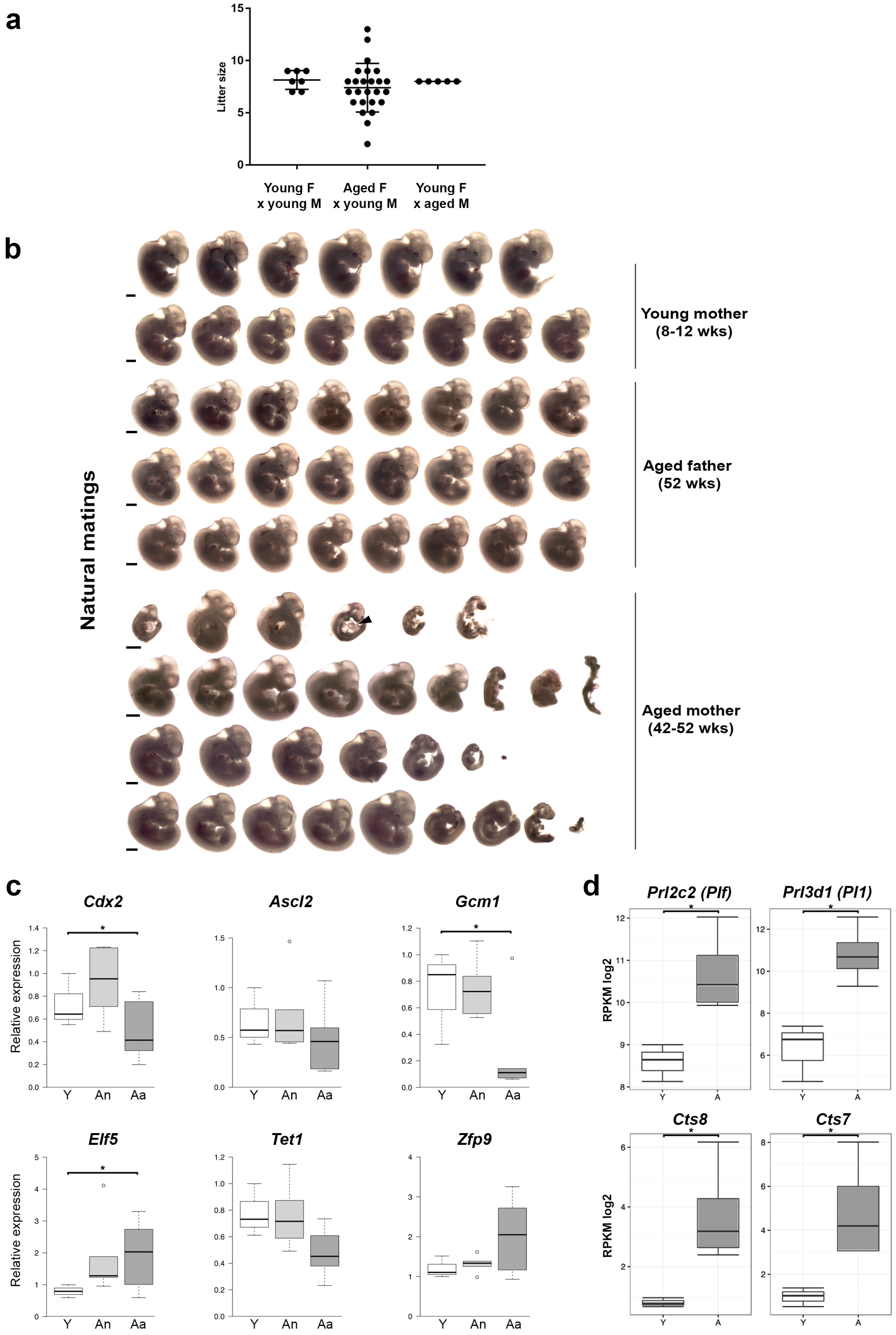

Supplementary Figure 1. Impact of maternal and paternal age on development. (a) Graph depicting the average litter size from different combinations of natural matings.

Each data point represents one litter. Scale bars: 1 mm. **(b)** Morphological analysis of E.115 embryos obtained from young (8-12 weeks old) females compared to embryos developed under the influence of aged (52 weeks old) C57BL/6 males or aged (42-52 weeks old) C57BL/6 females. Each row depicts one entire litter. Arrowhead indicates an example of an embryo with a pronounced pericardial edema. **(c)** Additional RT-qPCR analysis of placentas developed in young control (“Y”, n = 3) and aged (“A”) females divided into grossly normal (“An”; n = 6) or abnormal (“Aa”; n = 6). Markers analyzed encompass the trophoblast stem cell genes *Cdx2*, *Elf5* and *Tet1*, spongiotrophoblast and trophoblast giant cell marker *Ascl2* and *Zfp9*, and placental labyrinth expressed gene *Gcm1.* Data are represented as mean ± S.E.M. *= $p<0.05$ (ANOVA with Holm-Bonferroni’s post-hoc test). **(d)** Additional verification of up-regulated trophoblast giant cell marker gene expression in placentas of aged females determined by RNA-seq. Normalised read counts expressed as mean ± S.E.M. *= $p<0.05$ (two-tailed *t*-test).

a

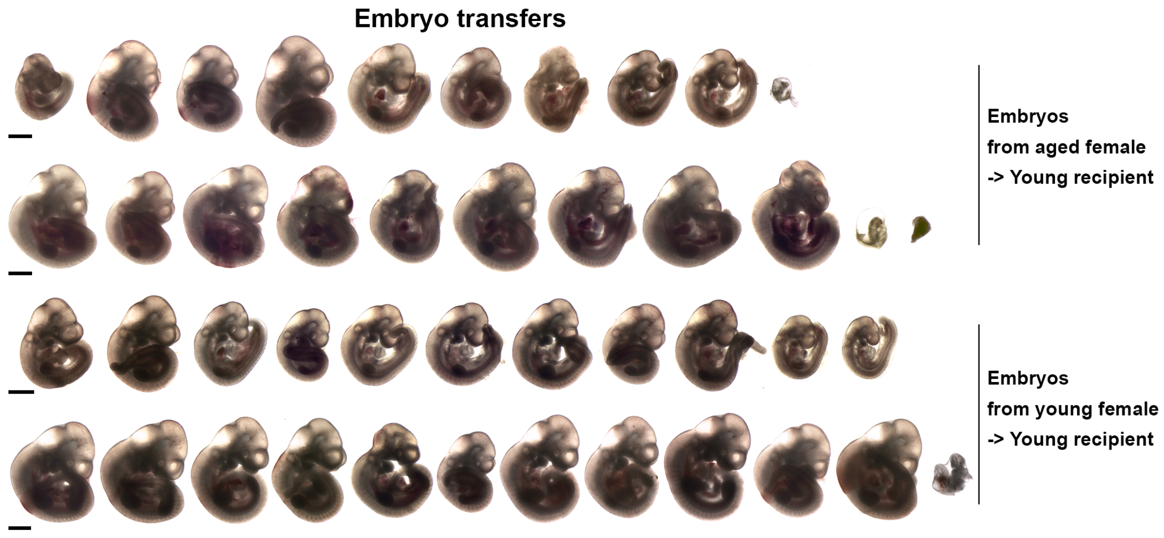

b

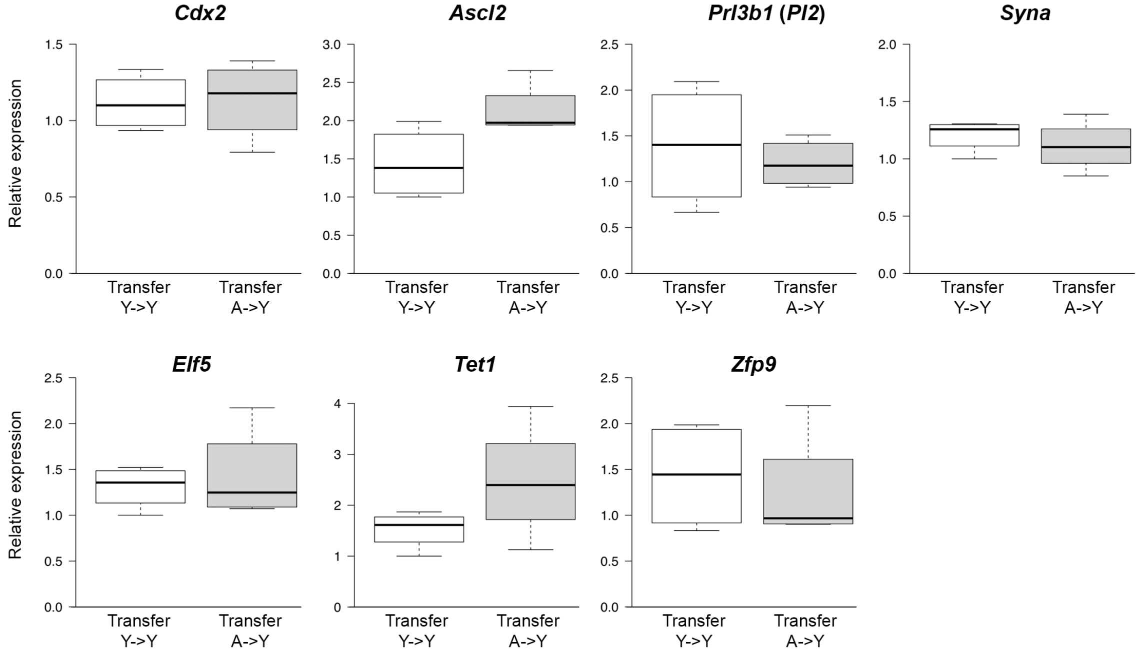

Supplementary Figure 2. Embryo transfer into young recipients rescues developmental defects. (a) Additional litters obtained from embryo transfers from aged to young (A->Y) recipient females, or from young to young (Y->Y) females as controls. Note the homogeneity of developmental progression of embryos derived from aged females developing in young females, compared to the litters shown in Fig. 1a and Suppl. Fig. 1b. Scale bars: 1mm. **(b)** RT-qPCR analysis of trophoblast markers in placentas of Y->Y and A->Y transfer conceptuses. No differences in expression levels are observed for any of the

genes tested (*t*-test), corroborating that placental development proceeds normally in A->Y transfer conceptuses. Data are mean ± S.E.M.

a

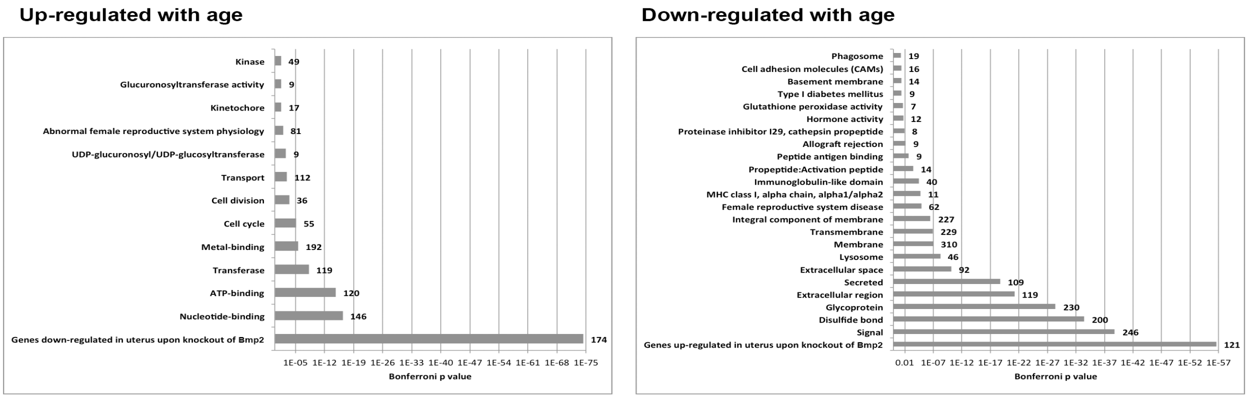

b

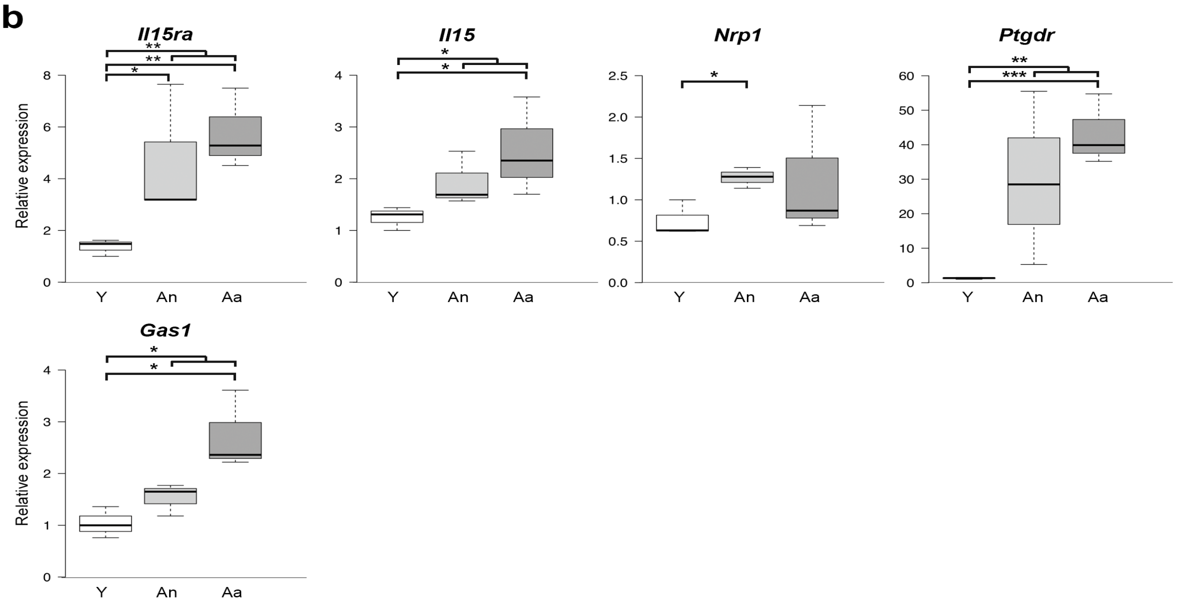

Supplementary Figure 3. Transcriptomic changes in E11.5 deciduas from young and aged females. (a) Gene ontology and gene set enrichment analysis, using the MSigDB database, performed separately on up- and down-regulated genes between E11.5 deciduas of conceptuses developed in aged and young females, as shown in Fig. 3a. **(b)** Additional validation of genes that were found up-regulated in the RNA-seq data: *Il15ra*, *Il15*, *Nrp1*, *Ptgdr* and *Gas1* were confirmed to be more highly expressed in deciduas of conceptuses developed in aged females (An, Aa) compared to those developed in young females (Y). Samples are as in Fig. 1c, data are mean ± S.E.M. *= p<0.05; **= p<0.01; ***= p<0.001 (ANOVA with Holm-Bonferroni's post-hoc test).

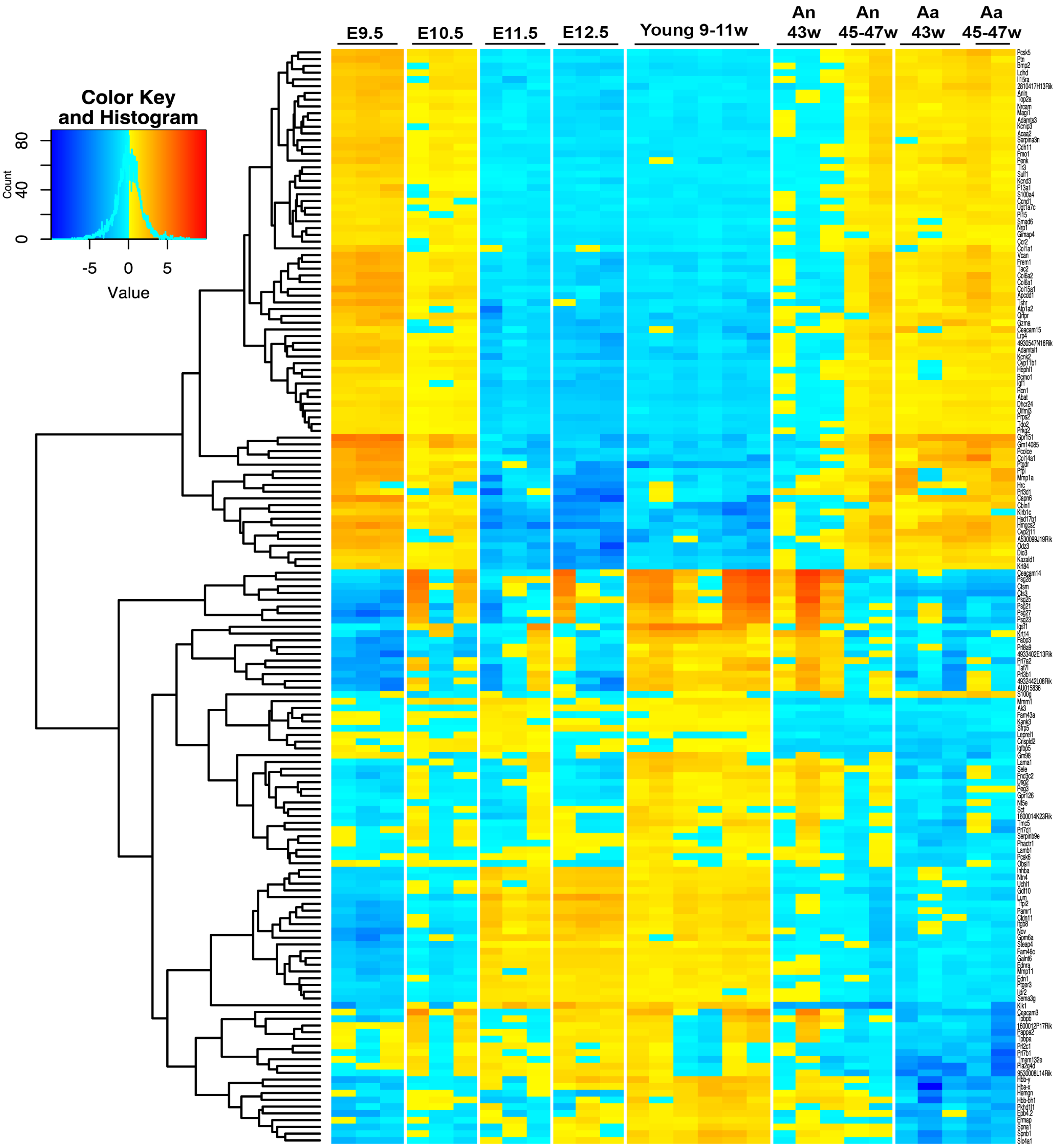

Supplementary Figure 4. Heatmap of differentially expressed genes integrated into developmental time course.

Heatmap showing differentially expressed genes from E9.5, E10.5, E11.5, E12.5 deciduas of pregnancies developed in young control B6 females that were integrated with the samples collected at E11.5 as shown in Fig. 3. Three independent deciduas from 3 litters each were dissected and sequenced for every developmental time point. Note that the gene expression profiles of E.11.5 aged deciduas resemble that of earlier developmental time points (E.9.5-E10.5) of samples from young females.

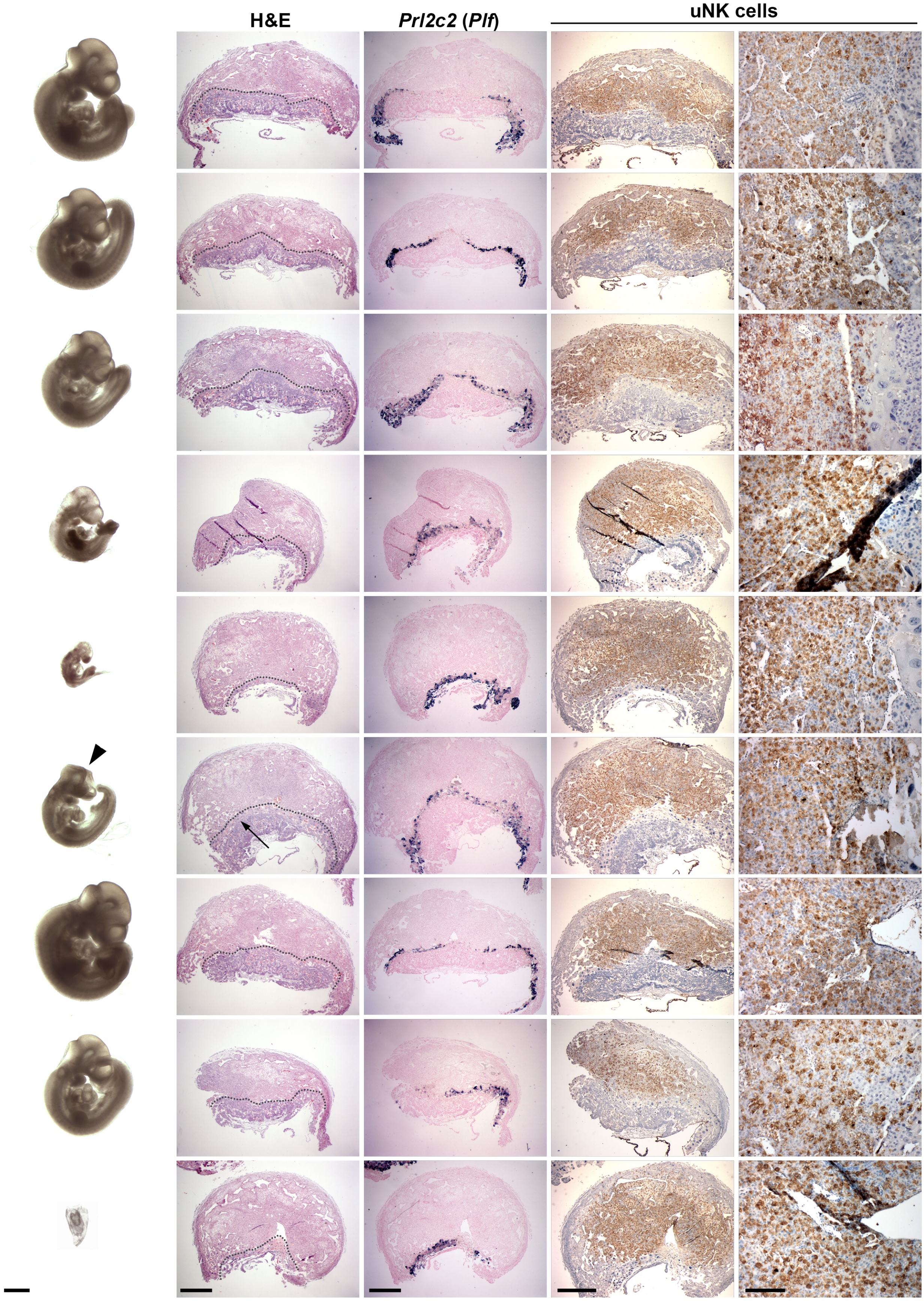
H&E
Prl2c2 (Plf)
uNK cells

Supplementary Figure 5. Comparative embryo and placenta development of a litter developed in an aged (54-wk old) female. Morphology of E11.5 embryos and their corresponding placentas, assessed histologically by H&E staining, *in situ* hybridisation for the trophoblast giant cell marker *Prl2c2* (*Plf*) and by staining for uNK cells using the *Dolichus biflores* agglutinin. As in Fig. 1, growth-retarded and/or abnormal embryos are associated with defective placentas, insofar as the trophoblast portion is severely under-developed (the dotted line indicates the boundary between the fetal trophoblast compartment and the maternal decidua) or the main direction of placentation is off-centre (arrow). Scale bars: 1st-4th column: 1 mm; 5th column: 200 µm.

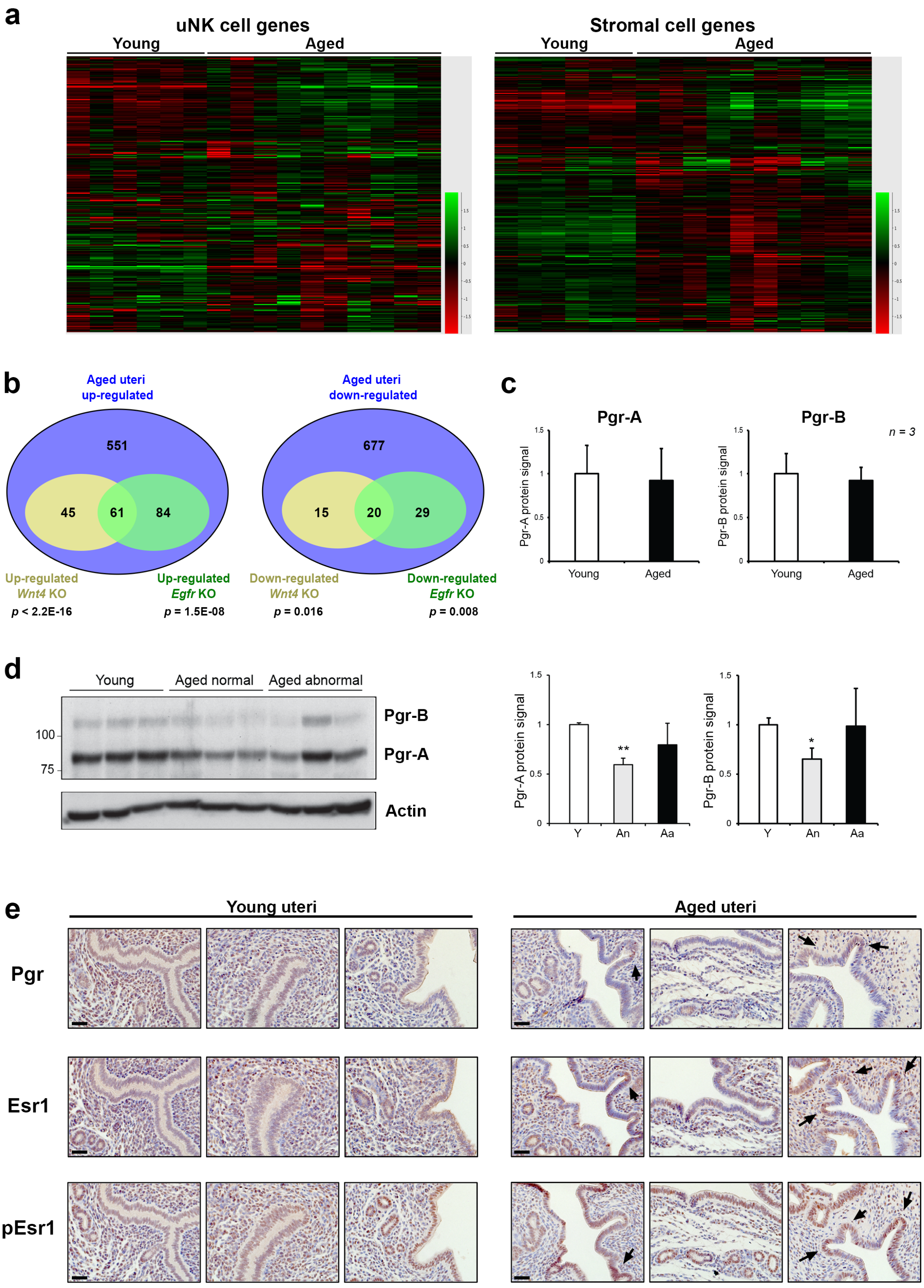

Supplementary Figure 6. Altered stromal cell response in aged females I. (a) Heatmap depicting the expression profiles of genes identified as characteristic of uNK or decidual stromal cells[1] in deciduas from young or aged females. The stromal cell signature

distinguishes young from aged samples more clearly than the uNK cell signature, indicating that stromal cells are more severely affected in aged deciduas **(b)** Venn diagrams of genes commonly up- and down-regulated during decidualisation in knockouts for *Egfr*, *Wnt4* and aged E3.5 uteri. **(c)** Progesterone receptor (Pgr) isoform -A and -B expression levels in uterine stromal cells from young and aged females, determined by Western blot. Uterine stromal cells were isolated from E3.5 young and aged mice, grown *in vitro* for 2 days and analysed. Graphs show Pgr protein signal intensity normalised against Actin and relative to young mice (mean ± S.E.M., n = 3). **(d)** Western blot for Pgr on E11.5 deciduas from young and aged females, the latter grouped into those that were attached to a grossly normal embryo (“Aged normal”, “An”) and those associated with an overtly abnormal embryo (“Aged abnormal”, “Aa”). Graphs show Pgr protein levels normalised to actin and relative to young mice (mean ± S.E.M., n = 3). *= $p<0.05$; **= $p<0.01$ (ANOVA with Holm-Bonferroni’s post-hoc test). **(e)** Histological examination of representative E3.5 uteri from young and aged mice (both mated with vasectomised males) and stained for Pgr, Esr1 and pEsr1. Arrows show mosaic staining pattern observed in the luminal epithelium of aged mice. Scale bars: 100 µm.

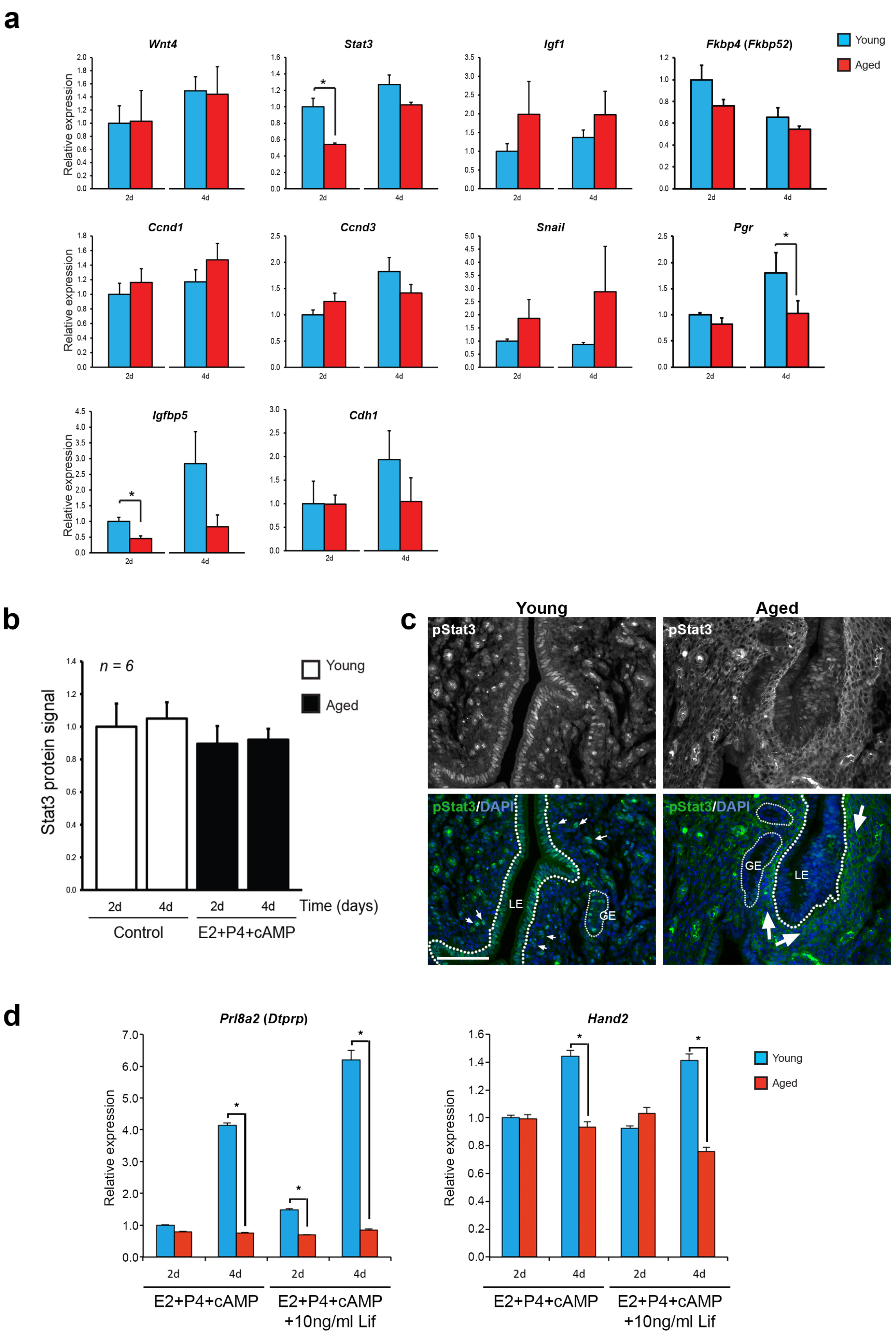
a
Wnt4
Stat3
Igf1
Fkbp4 (Fkbp52)
Young
Aged
Ccnd1
Ccnd3
Snail
Pgr
Igfbp5
Cdh1
Relative expression
2d
4d
b
n = 6
Young
Aged
Stat3 protein signal
2d
4d
Time (days)
Control
E2+P4+cAMP
c
Young
Aged
pStat3
pStat3/DAPI
LE
GE
d
Prl8a2 (Dtprp)
Hand2
Relative expression
E2+P4+cAMP
E2+P4+cAMP +10ng/ml Lif

Supplementary Figure 7. Altered stromal cell response in aged females II. (a) Additional RT-qPCR analyses of genes with key roles in decidualisation in stimulated decidual stromal cells. *= p<0.05 (n = 3, pairwise one-tailed t-test) **(b)** Western blot quantification of Stat3 protein signal in stimulated decidual stromal cells of young and aged females. Graph shows Stat3 protein levels normalised to Actin and shown relative to levels in 2d young mice (n = 6). **(c)** Immunofluorescence staining for pStat3 on E3.5 uteri of young and aged females. The dotted lines demarcate the boundary between luminal (LE) or glandular (GE) epithelium and the stromal cell compartment. Small arrowheads in the young sample highlight nuclear pStat3 staining in stromal cells. Large arrowheads in the aged sample point to the striking exclusion of pStat3 from nuclei of stromal cells. Nuclear pStat3 is also markedly reduced in GE and LE cells of aged uteri. Scale bar: 100 µm. **(d)** *In vitro* decidualisation of endometrial stromal cells as in Fig. 6e in standard conditions (E2+P4+cAMP) and in the presence of added Lif as activator of Stat signalling. *= p<0.05 (n = 3; ANOVA with Holm-Bonferroni's post-hoc test). All data are mean ± S.E.M.

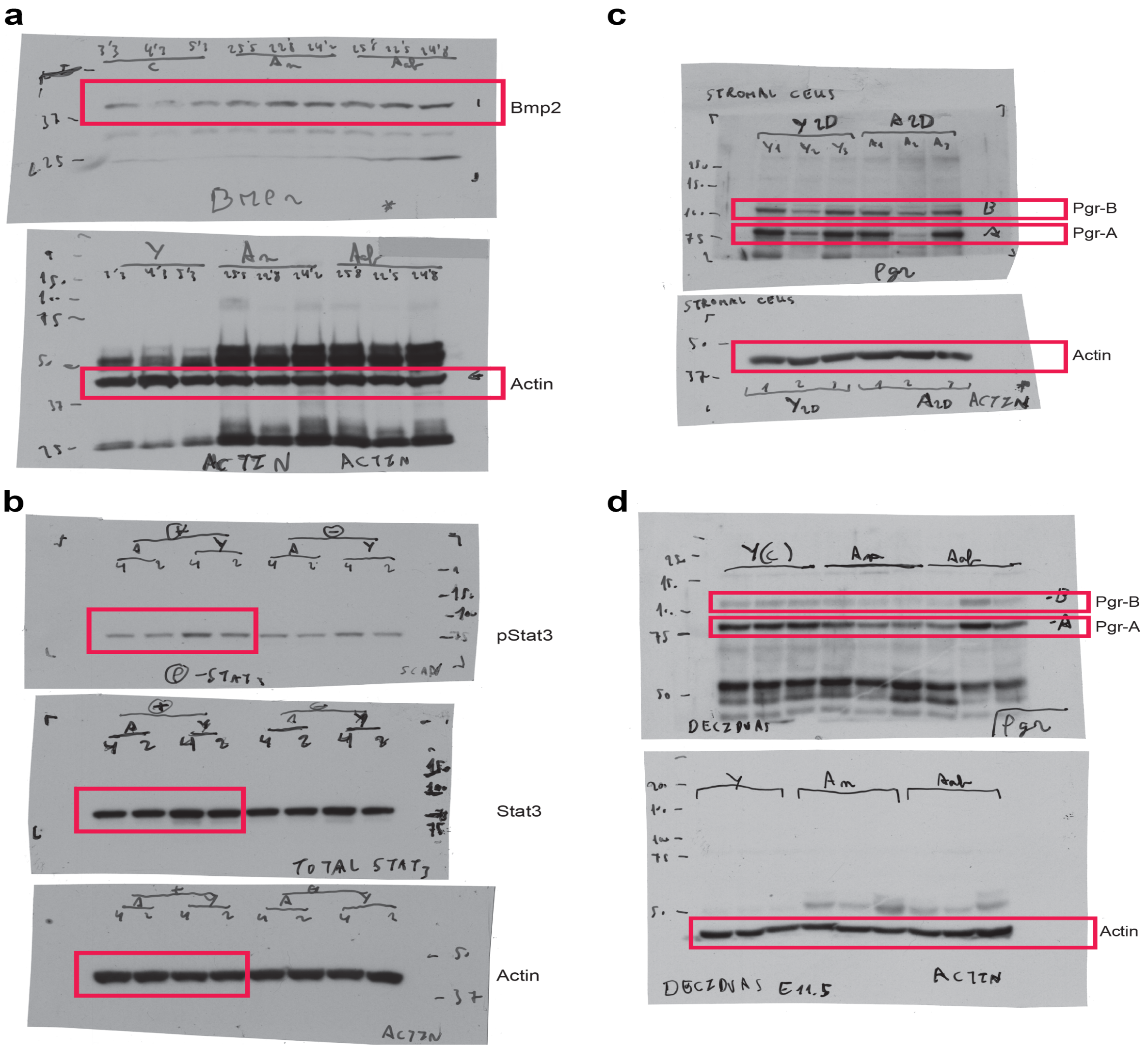

Supplementary Figure 8. Primary scans of Western blots. (a) Primary scans of Western blots as in Figure 3c. **(b)** Primary scans of Western blots as in Figure 6f and Supplementary Figure 7b. **(c)** Primary scans of Western blots as in Supplementary Figure 6c. **(d)** Primary scans of Western blots as in Supplementary Figure 6d.

Supplementary Table 1. qPCR primer sequences.

Gene	Sequence	
Ascl2	F	AGCCCGATGGAGCAGGAG
	R	CCGAGCAGAGGTCAGTCAGC
Bmp2	F	CCTGAAGCAGAGACCCACCC
	R	CTGGAAGTTCCTCCACGGCT
Ccnd1	F	CAGACCTTTGTGGCCCTCTGT
	R	GTTGTGCGGTAGCAGGAGAGG
Ccnd3	F	GCTCCAACCTTCTCAGTTGC
	R	AGCTAAGCAGCAAGCAAAGC
Cdh1	F	GAACGGTGGTCAAAGAGCCC
	R	CGAGTCACTTCCGGTCGGG
Cdx2	F	AGTGAGCTGGCTGCCACACT
	R	GCTGCTGCTGCTTCTTCTTGA
Ctsq	F	AATTGGCTATGGTTATGTGGGA
	R	TCACACAGTAGGGTATTGGG
Elf5	F	ATTCGCTCGCAAGGTTACTCC
	R	GGATGCCACAGTTCTCTTCAGG
Eomes	F	TCGCTGTGACGGCCTACCAA
	R	AGGGGAATCCGTGGGAGATGGA
Esrrb	F	AGTACAAGCGACGGCTGG
	R	CCTAGTAGATTCGAGACGATCTTAGTCA
Fkbp4 (Fkbp52)	F	GTCTGGACCGCAAGGACAAA
	R	ATGTGGCACACTTCCCCCAC
Gas1	F	CGAATCGGTCAAAGAGAACA
	R	GCTCGTCGTCATATTCTTCG
Gcm1	F	ACCCCTGAAGCTTATTCCCT
	R	TCGCCTTTGGACTGGAAA
Gdf10	F	ACAACCGAAGAGGTGCTCCA
	R	GAAGGCGGCTGTGAGGATCA
Hand2	F	CCAGCTACATCGCCTACCTC
	R	CTTGTCGTTGCTGCTCACTG

Hoxa10	F	CTGTCTCCAGCCCCTTCAGAAA
	R	AGTTGGCTGCATTTTCGCCTT
Igf1	F	CGATGGGGAAAATCAGCAGCC
	R	GCAGAGCGCCAGGTAGAAGA
Igfbp5	F	GCCCAACTGTGACCGCAAAG
	R	CACCAGCAGATGCCACGTTT
Il15	F	TCCACCTTGACACATGGCCC
	R	GCTTCCAGCTGCCATCCATC
Il15ra	F	TCCTCTTGGTTGGTGCAGGG
	R	GGTTTCCACCTCAACACGGC
Nr2f2 (Coup-tfII)	F	GCCAGTACTGCCGCCTCAA
	R	CAAACTGCCCGTGGGTAGGC
Nrp1	F	CCAAGCTCCGGAACCCTACC
	R	AAGGGCCCTGAAGACACCAC
Pgr	F	GGTGGGCCTTCCTAACGAG
	R	GACCACATCAGGCTCAATGCT
Prl3b1 (Pl2)	F	GCACTCGGGGAACAGCAGCC
	R	ACTGCCAGCAACAGGAGTGCC
Prl2c2 (Plf)	F	AACGCAGTCCGGAACGGGG
	R	TGTCTAGGCAGCTGATCATGCCA
Prl8a2 (Dtprp)	F	CAAACCCACCAGCTCATGGAC
	R	AGGAGTGATCCATGCACCCATAA
Ptgdr	F	CACCATGTGTTCCCTGCCTT
	R	GAAACGCAAGGCTTGGAGGT
Ptger3	F	TGAACCAGATCTTGGATCCCTGG
	R	GGTTGTTCATCATCTGGCAGAACTT
Sdha	F	TGGTGAGAACAAGAAGGCATCA
	R	CGCCTACAACCACAGCATCA
Sfrp5	F	TTCCCCCTGGACAACGACCT
	R	CGCTGTGCTCCATCTCACACT
Snail	F	TGCACGACCTGTGGAAAGGC
	R	AAGCACGGTTGCAGTGGGAG
Stat3	F	CTTGTCTACCTCTACCCCGACAT

	R	GATCCATGTCAAACGTGAGCG
Syna	F	CCTCACCTCCCAGGCCCCTC
	R	GGCAGGGAGTTTGCCCACGA
Synb	F	TCCGGAAAGGGACCTGCCCA
	R	CAGCAGTAGTGCGGGGTGCC
Tet1	F	GAGCCTGTTCCTCGATGTGG
	R	CAAACCCACCTGAGGCTGTT
Tpbpa	F	ACTGGAGTGCCCAGCACAGC
	R	GCAGTTCAGCATCCAACTGCG
Wnt4	F	CTCCTCGTCTTCGCCGTGTT
	R	GAGATGCTGCCCACCGATGA
Zfp9	F	ACCAGGAAACACACACCAAGA
	R	CGGTGAGGGCTGACTTCTG

Supplementary References

1. Nelson, A. C., Mould, A. W., Bikoff, E. K. & Robertson, E. J. Single-cell RNA-seq reveals cell type-specific transcriptional signatures at the maternal-foetal interface during pregnancy. *Nat. Commun.* **7**, 11414 (2016).
